# Identification of a queen primer pheromone in higher termites

**DOI:** 10.1038/s42003-022-04163-5

**Published:** 2022-11-02

**Authors:** Klára Dolejšová, Jan Křivánek, Jitka Štáfková, Natan Horáček, Jana Havlíčková, Virginie Roy, Blanka Kalinová, Amit Roy, Pavlína Kyjaková, Robert Hanus

**Affiliations:** 1 Chemistry of Social Insects, Institute of Organic Chemistry and Biochemistry of the Czech Academy of Sciences, Prague, Czech Republic; 2 Faculty of Science, Charles University, Prague, Czech Republic; 3 Present Address: Université Paris Est Créteil, Sorbonne Université, Université Paris Cité, CNRS, INRAE, IRD, iEES Paris, Créteil, France; 4 Czech University of Life Sciences, Prague, Czech Republic

**Keywords:** Animal physiology, Entomology

## Abstract

It is long established that queens of social insects, including termites, maintain their reproductive dominance with queen primer pheromones (QPPs). Yet, the QPP chemistry has only been elucidated in a single species of lower termites. By contrast, the most diversified termite family Termitidae (higher termites), comprising over 70% of termite species, has so far resisted all attempts at QPP identification. Here, we show that the queen- and egg-specific sesquiterpene (3*R*,6*E*)-nerolidol acts as the QPP in the higher termite *Embiratermes neotenicus*. This species has a polygynous breeding system, in which the primary queen is replaced by multiple neotenic queens of parthenogenetic origin. We demonstrate that (3*R*,6*E*)-nerolidol suppresses the development of these parthenogenetic queens and thus mimics the presence of mature queen(s). It acts as an airborne signal and may be used to optimize the number of queens, thus being the key regulatory element in the special breeding system of *E. neotenicus*.

## Abstract (web summary)

A queen primer pheromone in higher termites is identified and shown to control the development of new queens in the colony, and acts as an airborne signal detected via olfaction.

## Introduction

How the division of labor between reproducing and helping castes of eusocial insects has evolved and how it is established and maintained in their colonies are the central questions of social insect biology. Current evidence suggests that the reproductive division of labor in socially advanced eusocial insects (most eusocial wasps, ants, termites, and eusocial bees) is controlled via queen-emitted chemical signals, generally referred to as queen pheromones^1–3^. The definition of queen pheromones encompasses a broad array of effects of these signals on the receivers^2^, allowing the queens to advertise their presence, to be physically identified by their nestmates, and ultimately to monopolize colony reproduction.

Queen pheromones can act as *releaser pheromones* by directly affecting the behavior of the nestmates towards the queen upon her physical recognition (tending, feeding, defensive and retinue behaviors). At the same time, they may alter the physiology and development of the receivers and make them forgo or postpone their own reproduction in favor of the reigning queen(s), acting thus as *primer pheromones*^1,2^. Because queen primer pheromones (QPPs) inhibit the reproductive potential of other females in the colony, they have traditionally been considered tools of manipulation or control over the subordinate colony members. From the modern perspective, QPPs are viewed as honest signals of the queen’s presence and quality, allowing the nestmates to choose between individual reproduction and indirect gains from helping^4–6^. QPPs evolved independently in individual eusocial lineages to supersede the ancestral situation characterized by physical conflicts among nestmates over the queen status, encountered in some socially primitive taxa^3,6,7^.

In spite of the vital importance of QPPs for social homeostasis and prevention of conflict over reproduction in social insect colonies, their chemistry has long remained unknown. For nearly half a century, the honeybee Queen Mandibular Pheromone, in part described in the 1960s, remained the only chemically characterized QPP^8^. Unambiguous chemical identifications in other social insects only took place in 2010 and later, when the role of QPPs was ascribed to cuticular hydrocarbons (CHCs) on the queen’s body surface (or also on queen-laid eggs) in several species of ants and wasps and a bumblebee^9–13^, suggesting that multiple independent origins of eusociality within Hymenoptera were accompanied by co-options of a conserved ancestral CHC signaling system for the new role in queen fertility signaling^2,14^. By coincidence, in 2010 the first (and so far, the only) QPP was characterized in the lower termite *Reticulitermes speratus*^15^, indicating that termite QPPs may be chemically very different from CHCs (see below).

Most termite colonies are established by a pair of winged primary reproductives (primary king and queen), who may remain the only reproducing individuals in the colony throughout its life cycle. However, in many species an alternative reproductive phenotype exists, i.e. neotenic reproductives. These can develop from male and female immatures (larvae, workers, nymphs) as replacement kings or queens after colony orphaning, or as supplementary kings or queens in the presence of other reproductives^16^. A special context of neotenic formation has been described in three lower termites of the genus *Reticulitermes* (Rhinotermitidae)^17–19^ and several higher termites (Termitidae) from subfamilies Termitinae^20,21^ and Syntermitinae^22,23^. In these species, the founding primary queen prematurely dies, but before doing so, she produces a generation of female nymphs via thelytokous parthenogenesis. These nymphs develop into neotenic queens (NQs), replace the foundress, and reproduce in a polygynous breeding system with the founding primary king. At the same time, they also give rise asexually to subsequent generations of NQs, which supplement the pool of queens. Hence, the systematic production of NQs is the keystone of this unique reproductive strategy dubbed asexual queen succession (AQS).

Potential access of multiple colony members to neotenic reproductive options requires an effective mechanism by which the reigning queen(s) and king(s) advertise their presence and control the formation of neotenics. It has long been known that queens and kings do so via chemical cues which comply with the definition of primer pheromones^24–26^. Observations of king- and/or queen-specific profiles of CHCs in several termite species indicated that convergently with eusocial Hymenoptera, these non-volatile cues may be involved in fertility signaling^27,28^. CHCs were recently shown to act as releaser pheromones responsible for king and queen physical recognition^29,30^, and pieces of indirect evidence suggest that they may also act as QPPs, inhibiting neotenic formation^31^. Nevertheless, the only termite QPP was identified in 2010 in *Reticulitermes speratus* (Rhinotermitidae) as a volatile blend of *n*-butyl-*n*-butyrate and 2-methylbutan-1-ol^15^. The pheromone is secreted by NQs and inhibits the formation of further NQs from workers. The pheromone is also emitted by eggs, thus providing complementary information on the presence and quality of the queen(s). It also has a releaser function and serves as an attractant of the nestmates, regulates the oviposition rate in other queens^32^, and promotes the production of salivary lysozyme used by workers for egg protection^33^. Last but not least, it has antifungal properties against a specialized parasitic fungus^34^. This multifunctionality is a neat example of chemical parsimony, suggesting that antifungal defense was the primary role of the volatile blend, which only secondarily became the “central signal” ensuring colony homeostasis^35^.

Discovery of the QPP in *R. speratus* prompts questions about termite queen pheromones and queen pheromone communication of social insects in general, especially with respect to two conspicuous aspects: (i) the first identified termite QPP uses very different chemistry from that of QPPs in the honeybee and other Hymenoptera, and (ii) the first identified termite QPP is composed of highly volatile components, unlike the contact CHC cues in most eusocial Hymenoptera, seemingly to facilitate the spread of the signal across the large termite nests. Both these characteristics call for a comparison of different termite species to detect commonalities and idiosyncrasies in the QPP communication system of different social insect taxa. This perspective is particularly appealing for higher termites, the most diversified termite lineage (comprising 70% of living termite species^36^), whose colonies may contain up to millions of inhabitants. Himuro et al.^37^ reported phenylethanol as a queen-specific volatile of *Nasutitermes takasagoensis* (Nasutitermitinae), and we later identified the macrolide (5*Z*,9*S*)-tetradec-5-en-9-olide in queens and eggs of *Silvestritermes minutus* (Syntermitinae)^38^ and the sesquiterpene alcohol (3*R*,6*E*)-nerolidol in queens and eggs of four other Syntermitinae^39^. Yet, the biological role of these queen pheromone candidates could not be rigorously studied due to difficulties in maintaining colonies of higher termites in captivity and in experimental groups.

In the present study, we search for the QPP in the higher termite *Embiratermes neotenicus* (Syntermitinae). This species is among the rare termites having the AQS breeding system; the founding primary queen is replaced early in the colony cycle by tens to hundreds of NQs, which in turn produce further generations of parthenogenic queens^23^. The polygynous AQS breeding system calls for the presence of a control mechanism that would ensure the correct timing of development of NQs from parthenogenetic nymphs upon the death (or senescence) of the foundress and later regulate the numbers of supplementary NQs according to the colony population size and the fitness of the reigning queen(s). Our observations on *E. neotenicus* and the related AQS species *Silvestritermes minutus* revealed that throughout the year, a pool of units to tens (*S. minutus*) or tens to hundreds (*E. neotenicus*) of parthenogenetic female nymphs of the fourth developmental stage (NY4**♀**) is present in most colonies. When isolated from queens, many of these NY4**♀** rapidly, within a few days, molt into NQs^22,23^. Therefore, NY4**♀** can be considered arrested in their developmental trajectory, awaiting the appropriate moment to develop into NQs, and may thus be a potential target of the QPP control. We previously reported that primary queens and NQs of *E. neotenicus* emit significant quantities of enantiomerically pure (3*R*,6*E*)-nerolidol (hereafter referred to as RNERO) to the headspace^39^ and that the compound is also present in washes of eggs while being absent in all other castes and stages in the colony, including NY4**♀** and young subfertile NQs. Because of its queen- and egg-specific distribution, RNERO is a suitable candidate for a QPP function. Here, we test its potential role as QPP in a series of short-term experiments performed with freshly collected *E. neotenicus* colonies.

A remarkable characteristic of the AQS breeding system is that the NQs predominantly develop from NY4**♀** of parthenogenetic origin while the sexually produced female nymphs occurring in colonies prior to dispersal flights mostly develop into winged dispersers. Even though the proximate mechanisms of these developmental priorities remain unknown and are broadly discussed^40–43^, the differential developmental readiness of female nymphs according to their genetic origin is vital for the function of AQS systems. Here, we address this question by including in our experiments both colonies having relatively small numbers of mostly female NY4 presumed to be parthenogenetic as well as colonies nearing dispersal and containing hundreds to thousands of male and female nymphs of sexual origin. We test the readiness of female nymphs from both colony types to become NQs in the presence or absence of NQs and RNERO. In queen inhibition bioassays, we assess the genetic origin of nymphs and newly developed NQs by genotyping them at previously developed microsatellite markers.

We show that RNERO inhibits the formation of new NQs from NY4**♀** of parthenogenetic origin and thus mimics the effect of live NQs in experimental groups. By contrast, in queenless conditions or in absence of RNERO, the parthenogenetic NY4**♀** respond by rapid differentiation into new NQs unlike the sexually produced NY4**♀** from colonies nearing dispersal. We prove that RNERO acts as an airborne signal, and shows selective olfactory preference for RNERO by NY4**♀**, compared to its opposite (*S*) enantiomer and related sesquiterpenoids, putting forward the role of RNERO as a signal mediated via olfaction. These results confirm RNERO as the second QPP identified in termites and the first to be described in higher termites. At the same time, they reveal that the two termite QPPs identified so far present fundamentally different chemistries, suggesting that the array of termite QPPs can be unexpectedly diverse.

## Results

### Readiness of nymphs to develop into neotenic queens

**Fig. 1:**
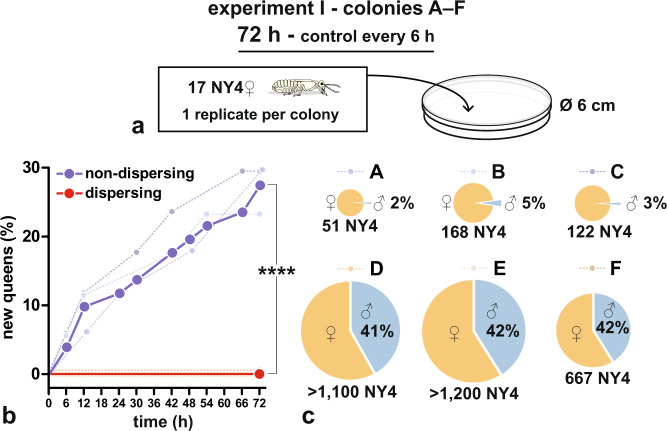
Readiness of female nymphs to develop into neotenic queens. Groups of 17 NY4**♀** from three non-dispersing (A–C) and dispersing (D–F) colonies were observed for 72 h and numbers of new NQs in the groups scored. **a** Experiment design. **b** Dashed curves indicate cumulative numbers of new NQs in individual colonies. Bold curves indicate total cumulative numbers of new NQs in non-dispersing (blue) and dispersing (red) colonies, compared using Mantel–Cox test (*χ*^2^ = 16.13, df = 1, *p* < 10^−4^). **c** Pie charts show numbers of NY4 found in the six colonies (pie size) and proportions of males among NY4.

**Fig. 2:**
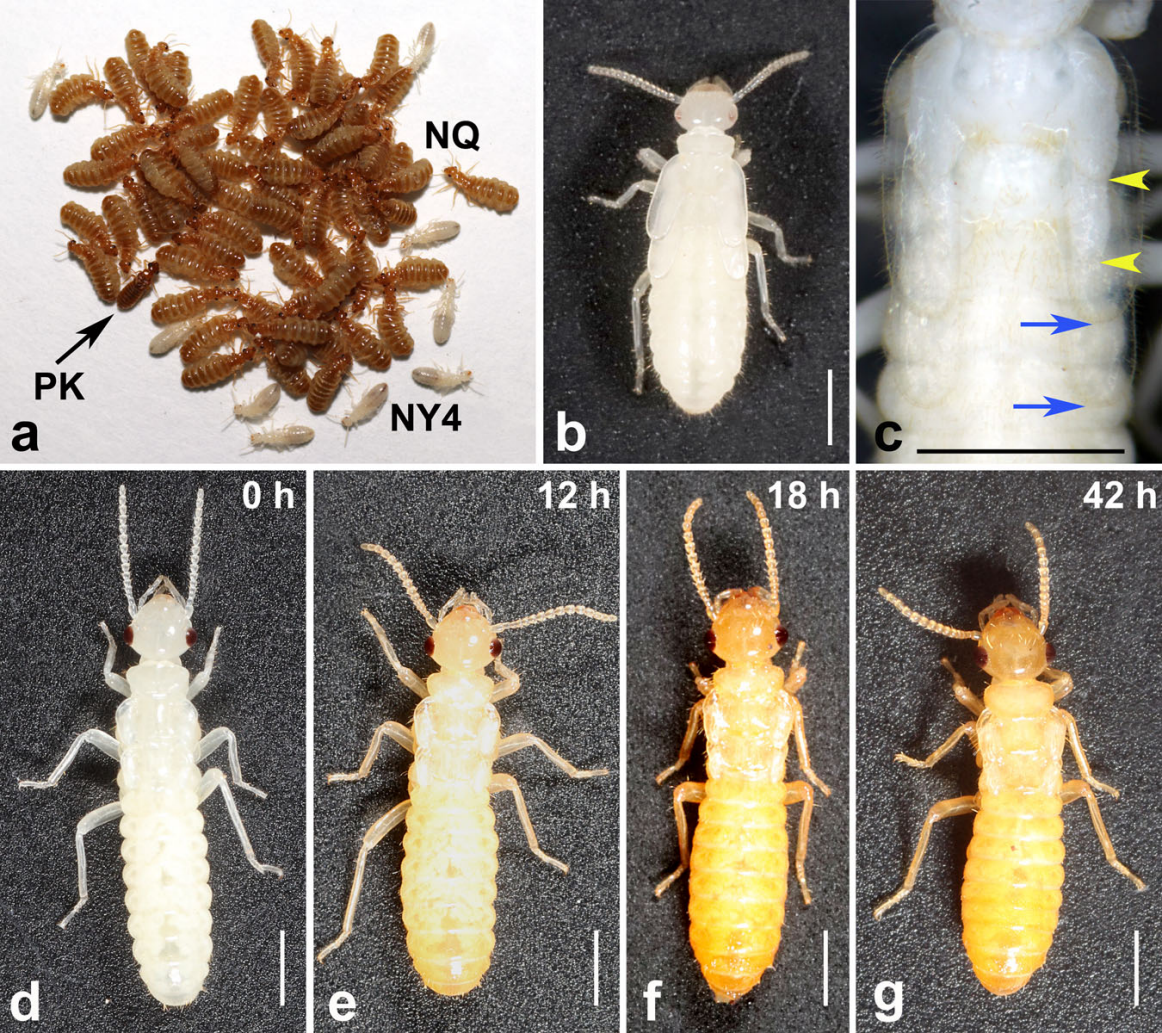
Development and maturation of neotenic queens in *E. neotenicus*. **a** Primary king (PK) accompanied by a group of mature neotenic queens (NQ) and female nymphs (NY4). **b** NY4**♀** from a non-dispersing colony. **c** Pharate neotenic queen before shedding the cuticle of NY4**♀**. The tips of wing rudiments of future NQ are marked with arrowheads, tips of NY4**♀** wing pads are marked with arrows. Scale bar indicates 1 mm. **d**–**g** Neotenic queen 0, 12, 18, and 42 h after molting from NY4**♀**.

In the initial experiment I, we tested the readiness of NY4**♀** to develop into NQs and possible differences between the readiness of NY4**♀** from non-dispersing colonies (expected to be predominantly parthenogens) and NY4**♀** from large dispersing colonies (dominantly nymphs of sexual origin). To do so, we isolated groups of 17 NY4**♀** from six different colonies (A–F), three of them representing typical non-dispersing colonies (low hundreds of NY4**♀**, male nymphs being rare), while the other three were nearing dispersal and contained high hundreds to thousands of NY4 with a substantial proportion of males (41–42%) (Fig. 1). NY4**♀** from each of the three non-dispersing colonies started to differentiate into NQs soon after their isolation and reached the final counts of four to five NQs per one group within 72 h, i.e., 27% of all nymphs from non-dispersing colonies. By contrast, we did not observe any of the 51 nymphs from the dispersing colonies transform into NQ. The survival curves constructed from total cumulative counts of new NQs in dispersing vs. non-dispersing colonies were significantly different (Mantel–Cox test, *χ*^2^ = 16.13, df = 1, *p* < 10^−4^). The transformation of NY4**♀** into NQ and subsequent NQ maturation are documented in Fig. 2.

### Presence of RNERO in queens and eggs

Experiments II and III, testing the effect of NQs and RNERO on the development of new NQs, were performed on three colonies (G–I) having relatively small numbers of NY4 (<300), since NY4**♀** in such colonies responded to orphaning by differentiation into NQs unlike the nymphs from large dispersing colonies. During the establishment of queen inhibition bioassays, we sampled mature NQs, NY4**♀**, workers, soldiers, eggs, and primary king (when found) for chemical analyses to verify the production of RNERO by NQs and eggs. The sample set was later supplemented by young NQs which were obtained from the bioassays and subsequently kept in a colony fragment for 9 h, 10 or 14 days.

**Fig. 3:**
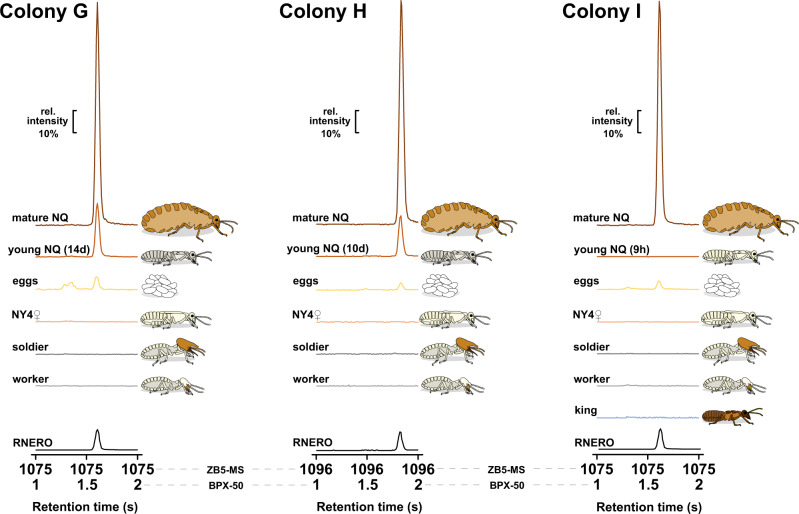
Mature NQs and eggs from the three colonies used in queen inhibition bioassays produce RNERO. Figure shows portions of 2D gas chromatograms of hexane washes of various available castes and life stages from colonies G–I, and of synthetic RNERO standard. Termites were sampled into identical amounts of solvent (30 µl/ind., 30 µl/approx. 50 eggs) directly from the colonies, except for young NQs, which were obtained in the bioassays and held in colony fragments for 9 h, 10, or 14 days. Figure depicts a portion of one modulation period (1–2 s) of the secondary column (BPX- 50) at the elution time of RNERO from the primary column (ZB5-MS). The shift in retention times of RNERO is due to the exchange of columns between analyses performed in 2021 (G, I) and 2022 (H).

Two-dimensional gas chromatography–mass spectrometry (GC×GC-MS) analyses confirmed that the extracts of mature NQs and eggs from the three colonies contained RNERO, as evidenced by characteristic retention parameters and MS fragmentation pattern (Fig. 3). By contrast, the peak of RNERO was absent in extracts of workers, soldiers, primary king (colony I) and NY4**♀**. We did not detect RNERO in freshly differentiated, non-physogastric and non-pigmented NQs 9 h after the molt from NY4**♀** (colony I), while pigmented but non-physogastric NQs 10 and 14 days after the molt already produced RNERO in significant quantities (colonies G and H). Full chromatograms and comparisons of MS spectra are given in Supplementary Figs. 1–4.

### NQs and RNERO inhibit differentiation of new NQs

**Fig. 4:**
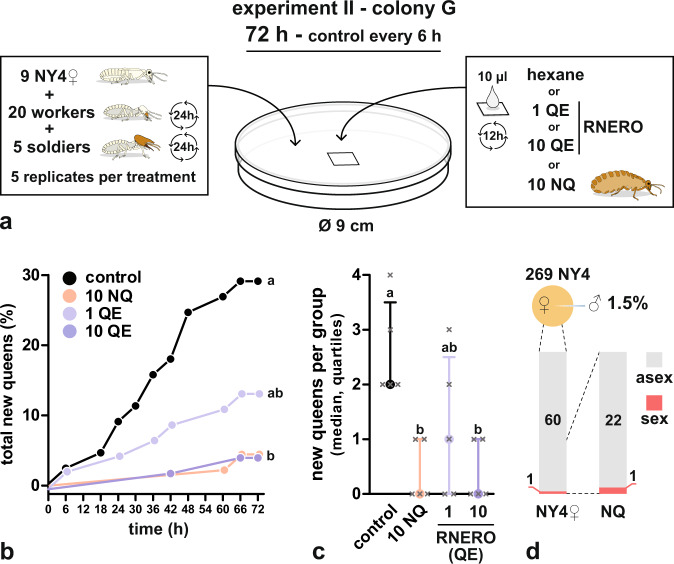
Presence of mature queens or RNERO inhibits the development of new queens from female nymphs. **a** Design of queen inhibition experiment with colony G. **b** Cumulative numbers of new NQs in solvent control and under different treatments. The highly significant overall difference among all curves (Mantel–Cox test, *χ*^2^ = 17.24, df = 3, *p* = 6 × 10^−4^) was mainly due to significant differences of 10NQ and 10QE treatments from the control. Different lowercase letters indicate significant differences at *α* = 0.05 corrected using Sidak correction for multiple comparisons (pairwise Mantel–Cox test comparisons). **c** Median numbers (and quartiles) of new NQs per one experimental group, showing significant overall differences (K–W rank-sum test, *H* = 10.24, groups = 4, *n* = 20, *p* = 1.67 × 10^−2^). Different lowercase letters indicate significant pairwise differences at *α* = 0.05 in Dunn’s Multiple Comparison Test. **d** Pie chart shows the number of NY4 found in the colony (pie size) and the proportion of males among NY4. Left column shows the proportion of sexually (sex) and parthenogenetically (asex) produced NY4**♀** in the genotyped subset of females that entered the experiment. Right column shows the proportion of sexually and parthenogenetically produced NQs that developed in the experiment.

In experiment II, we exposed groups of 9 NY4**♀** from colony G, accompanied by workers and soldiers, to four treatments: solvent control, 10 live mature NQs (10NQ), 1 and 10 queen headspace equivalents of RNERO (1QE and 10QE) for 72 h, with five replicate groups per treatment and a design allowing direct contact of the termites with NQs and the treated filter paper (Fig. 4a).

During those 72 h, 13 out of the 45 NY4**♀** in the solvent control groups differentiated into NQs (28.8%), 6 new queens were observed in 1QE treatment (13.3%) and 2 in the 10NQ and 10QE treatments (4.4%). Survival curves generated from cumulative counts of new NQs in individual treatments (Fig. 4b) were significantly different (Mantel–Cox test, *χ*^2^ = 17.24, df = 3, *p* = 6 × 10^−4^), especially due to differences between the control and 10NQ and 10QE treatments (*p* = 1.5 × 10^−3^ and 1.7 × 10^−3^, respectively, corrected *α*_(0.05)_ = 8.5 × 10^−3^).

Likewise, significant differences were observed when the variability among the five replicates was considered (K–W rank-sum test, *H* = 10.24, groups = 4, *n* = 20, *p* = 1.67 × 10^−2^), resulting especially from significant differences between the control (median = 2) and 10NQ and 10QE treatments (median = 0, Dunn’s multiple comparison test, adjusted *p* = 0.033 for both) (Fig. 4c). In brief, both stronger treatments (10NQ, 10QE) significantly reduced the numbers of new NQs, while the weaker treatment (1QE) revealed an intermediate effect, which did not differ significantly from other treatments and control.

### RNERO inhibits differentiation of new NQs as an airborne factor

**Fig. 5:**
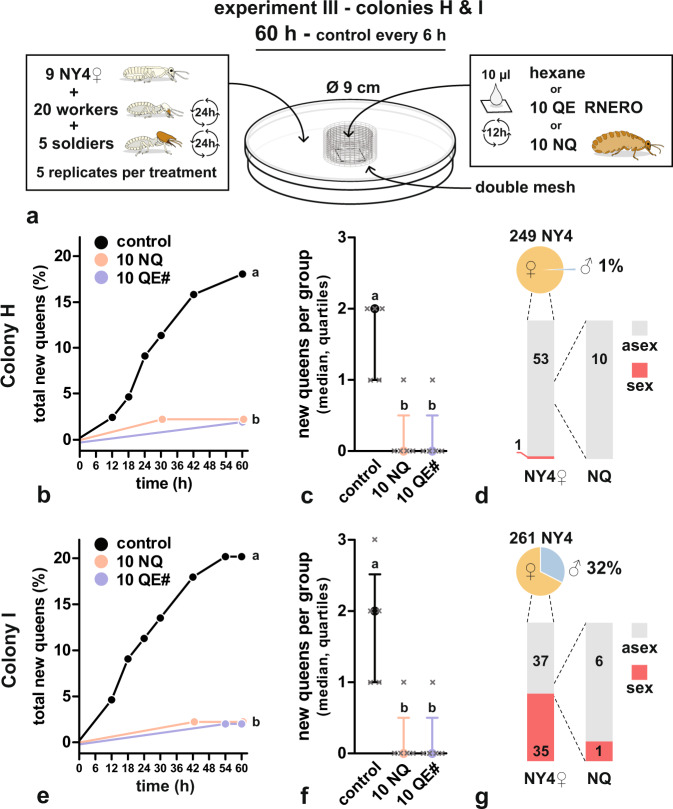
RNERO acts as an airborne signal and inhibits the development of new queens from female nymphs without physical contact with its source. **a** Design of queen inhibition experiment with colonies H and I. **b** and **e** Cumulative numbers of new NQs in solvent control and under different treatments. Highly significant overall differences among all curves (Mantel–Cox test; colony H: *χ*^2^ = 10.96, df = 2, *p* = 4.2 × 10^−3^; colony I: *χ*^2^ = 13.16, df = 2, *p* = 1.4 × 10^−3^) were due to significant differences of 10NQ and 10QE# treatments from the control. Different lowercase letters indicate significant differences at *α* = 0.05 corrected using Sidak correction for multiple comparisons (pairwise Mantel–Cox test comparisons of all curves). **c** and **f** Median numbers (and quartiles) of new NQs per one experimental group, showing significant overall differences (KW rank-sum test; colony H: *H* = 9.62, groups = 3, *n* = 15, *p* = 6 × 10^−3^; colony I: *H* = 9.56, groups = 3, *n* = 15, *p* = 6 × 10^−3^). Different lowercase letters indicate significant pairwise differences at *α* = 0.05 in Dunn’s Multiple Comparison Test. **d** and **g** Pie charts show the numbers of NY4 found in the colonies (pie size) and the proportion of males among NY4. Left columns show proportions of sexually (sex) and parthenogenetically (asex) produced NY4**♀** in the genotyped subset of females that entered the experiment. Right column shows the proportion of sexually and parthenogenetically produced NQs that developed in the experiment.

Based on the above observations we designed Experiment III with colonies H and I. The general design was identical to Experiment II (Fig. 5a). Termite groups were exposed for 60 h to three treatments, i.e., solvent control, 10 live mature NQs (10NQ) and 10 queen headspace equivalents of RNERO loaded on a piece of filter paper surrounded with a fine double mesh, preventing, in this case, the direct contact of the termites with the paper (10QE#).

While in solvent controls 8 (17.7%) and 9 (20%) new NQs developed during 60 h in colony H and I respectively, only a single NQ was retrieved in the 10NQ and 10QE# treatments (2.2%) in each colony (Fig. 5b, e). Survival curves of cumulative counts of new NQs were significantly different in both colonies (colony H: *χ*^2^ = 10.96, df = 2, *p* = 4.2 × 10^−3^, Fig. 5b; colony I: *χ*^2^ = 13.16, df = 2, *p* = 1.4 × 10^−3^, Fig. 5e) due to the significant differences between the control and both treatments (colony H: *p* = 0.0142 for control vs. 10NQ and 0.0133 for control vs. 10QE#; colony I: *p* = 7.1 × 10^−3^ for control vs. 10NQ and 6.9 × 10^−3^ for control vs. 10QE#; corr. *α* = 0.0169). When the variability among replicates was compared (Fig. 5c, f), significant overall statistics were also obtained (colony H: *H* = 9.618, groups = 3, *n* = 15, *p* = 6 × 10^−3^; colony I: *H* = 9.556, groups = 3, *n* = 15, *p* = 6 × 10^−3^), driven by the differences between the controls and the 10NQ and 10QE# treatments (adjusted *p* = 0.022 for both comparisons in both colonies).

### Genetic origin of NY4♀ and NQs in colonies G–I

To establish the genetic background of female nymphs in the colonies G–I used for queen inhibition bioassays, and to address the potential differential readiness of parthenogenetic vs. sexually produced NY4**♀** to develop into NQs, we genotyped subsets of workers (12–14 per colony), soldiers (9–12), NY4**♀** (38–65), and NQs newly molted in the experiments (7–23). We inferred the parental genotypes for each colony and deduced the genetic origin of NY4**♀** and NQs from the presence/absence of exclusive parental allele(s) in their genotypes. Genotypes recorded at the nine studied microsatellite markers in the 259 individuals are listed in Supplementary Table 1.

In agreement with our expectations based on the small number of NY4 (269) and low proportion of NY4 males (1.5%), colony G revealed to be a non-dispersing colony with only one out of 61 genotyped females (38 NY4**♀** + 23 NQs) being sexually produced (Fig. 4d). Likewise, all but one among 54 genotyped females (44 NY4**♀** + 10 NQs) were of parthenogenetic origin in colony H, having 249 NY4 and 1% of males (Fig. 5d). Out of the 72 successfully genotyped females (65 NY4**♀** + 7 NQs) from colony I, 37 were parthenogenetic and the remaining 35 sexually produced, suggesting a roughly balanced initial ratio of sexually vs. parthenogenetically produced NY4**♀** randomly selected for the inhibition bioassay (Fig. 5g). This indicates, together with the presence of 32% of males among NY4, that in spite of the relatively low total number of NY4 (261), typical of non-dispersing nests, colony I was in fact nearing a small dispersal event and the pool of parthenogens was in part diluted by sexually produced NY4**♀**. By contrast, among the 7 successfully genotyped NQs only one was of sexual origin.

In total, among all NY4**♀** randomly selected from their natal colonies, randomly attributed to one of the treatments in the queen inhibition bioassay and randomly selected for genotyping, only two sexually produced individuals developed into NQs out of 37 (5.4%). This contrasts with 60 new NQs that differentiated from 150 parthenogenetic NY4**♀** (25%) (Figs. 4d, 5d, g, Supplementary Table 1).

### Olfactory preference for RNERO by NY4♀

**Fig. 6:**
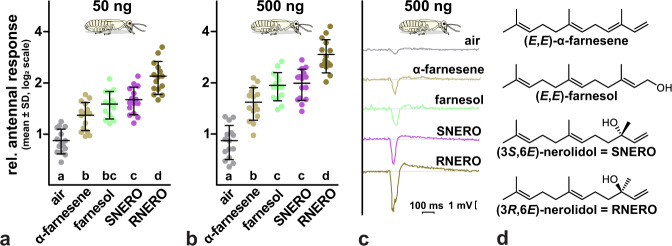
Olfactory responses of female nymphs show a significant preference for RNERO over its opposite enantiomer and related sesquiterpenoids. **a** and **b** Graphs show EAG responses (amplitude values) of NY4**♀** to air, RNERO, its opposite (*S*) enantiomer (SNERO) and two other structurally related sesquiterpenoids, (*E*,*E*)-α-farnesene and (*E*,*E*)-farnesol at doses of 50 ng (**a**) and 500 ng (**b**) of stimuli. The graphs show the means (±SD) of 15 independent stimulation series with randomized order of stimulations, related to responses to hexane stimulations; log_2_ scale is used. ANOVA test for both used doses revealed significant differences in antennal responses to different stimuli, with means ranked in the order: air < α-farnesene < farnesol < SNERO < RNERO (50 ng: *F*_4,70_ = 43.5, *p* < 10^−4^; 500 ng: *F*_4,70_ = 62.2, *p* < 10^−4^). Columns marked with different lowercase letters differed significantly at *α* = 0.05 when compared using Tukey’s multiple comparison test among all pairs of columns. Detailed test statistics are provided in the Supplementary Data 1 file. **c** EAG response traces showing one stimulation series using 500 ng of the compounds. **d** Structures of the tested compounds.

The results of queen inhibition bioassays suggested that RNERO acts as an airborne signal detected by olfaction. Therefore, we decided to test electroantennographic responses of NY4**♀** to RNERO and three structurally related sesquiterpenoids, i.e. (*E*,*E*)-α-farnesene, (*E*,*E*)-farnesol and (3*S*,6*E*)-nerolidol in order to address the potential olfactory preference for RNERO over related structures. Electroantennographic measurements revealed significant differences in antennal responses of NY4**♀** to the four sesquiterpenoids and the solvent control at both tested doses (50 ng: ANOVA, *F*_4,70_ = 43.46, *p* < 10^−4^; 500 ng: *F*_4,70_ = 62.18, *p* < 10^−4^) (Fig. 6a–c). At both doses, similar ranking of the mean responses was observed: control < (*E*,*E*)-α-farnesene < (*E*,*E*)-farnesol < (3*S*,6*E*)-nerolidol < RNERO, with most pairwise comparisons being significant at *α* = 0.05 [except for farnesol vs. (3*S*,6*E*)-nerolidol at both doses and (*E*,*E*)-α-farnesene vs. farnesol at 50 ng]. Detailed test statistics of all pairwise comparisons are provided in the Supplementary Data 1 file. The difference between the mean response to RNERO and (3*S*,6*E*)-nerolidol was strikingly larger than among other pairs of treatments, suggesting a strong stereoselective olfactory preference for RNERO over all other compounds including its opposite (*S*) enantiomer.

## Discussion

In this study, we show that RNERO suppresses the differentiation of new NQs from NY4**♀** of parthenogenetic origin and thus mimics the presence of mature NQs in experimental groups. By contrast, when liberated from exposure to RNERO or mature NQs, these parthenogenetic NY4**♀** rapidly respond by differentiation into new NQs, with up to almost 30% of NY4**♀** molting into NQs within 3 days. RNERO also effectively suppresses the development of new NQs in experiments preventing direct contact of the termites with its source, indicating its function as an airborne signal acting via olfaction. In line with these observations, electrophysiological experiments revealed a strong antennal response of NY4**♀** to RNERO and significant enantioselective preference for RNERO over its opposite (*S*) enantiomer and other structurally related acyclic sesquiterpenoids. Our results also showed that sexually produced NY4**♀**, present in colonies nearing dispersal, only rarely respond to orphaning or RNERO absence by differentiation into NQs.

**Fig. 7:**
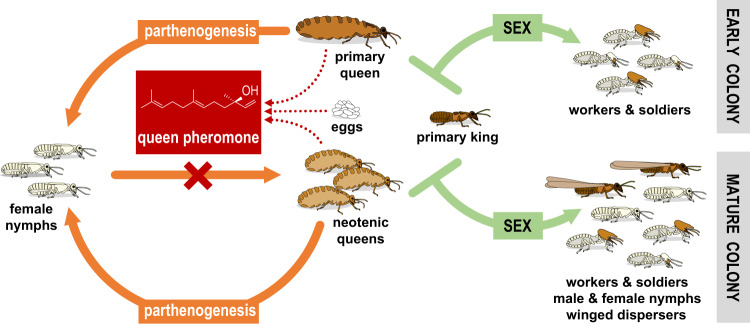
Proposed scheme of genetic and pheromonal regulation of the AQS breeding system in *E. neotenicus*. Based on the present results and our previous observations on the chemical ecology and breeding system of the species^23,39,44,58^.

Our observations lead us to conclude that RNERO acts as the queen pheromone by which the mature queen(s) inhibits the formation of new neotenic queens from parthenogenetic female nymphs. We showed here and previously^39^ that both the mature founding primary queen and mature NQs emit RNERO on the body surface and to the headspace. By doing so, the primary queen may delay the onset of the polygynous breeding system from à priori-produced parthenogens as long as she remains fertile and fit. Likewise, after the replacement of the foundress by multiple parthenogens, the queen pheromone secreted by NQs may delay the differentiation of new supplementary NQs. The same function can be ascribed to RNERO emitted by queen-laid eggs, which represent a direct indicator of queen fecundity. RNERO is the second functionally confirmed termite QPP, and the first to be characterized in higher termites. It may serve for the correct timing of primary queen replacement and optimization of the number of NQs according to the colony size and fitness of the reigning queen(s). Pheromone control over the development of new parthenogenetic queens is a key regulatory element in the AQS breeding system and a negative feedback complement of the genetic control via the production of sexual vs. parthenogenetic female nymphs (Fig. 7).

In *E. neotenicus*, we did not previously detect any sexually produced NQs across tens of colonies^23,44^. Indeed, our present experiments show that also under experimental conditions the NY4**♀** of sexual origin only rarely differentiate into NQs, irrespective of the presence or absence of the queen(s) or QPP in the experimental groups. Therefore, whatever the mechanism determining the developmental priorities of female nymphs, our experiments underline the adaptive significance of the combined (epi)genetic and pheromonal control over queen development in AQS breeding systems: while the sexually produced female nymphs are almost unreservedly channeled into winged dispersers to ensure the genetic diversity of future dispersing queens and prevent inbred mating with their father, the developmentally arrested parthenogenetic NY**♀** respond to the absence of QPP to allow an optimized number of non-dispersing parthenogens of the foundress for outbred mating with the primary king (Fig. 7).

The search for volatile queen pheromones in higher termites was motivated by the pioneering description of the first queen pheromone in the lower termite *R. speratus*^15^. Yet, while the chemical identifications of pheromone candidates were rather straightforward^37–39^, functional studies were hampered by the difficulties in performing bioassays with higher termites and keeping their colonies in captivity^38,39^. Our previous attempts to study the role of RNERO with *E. neotenicus* laboratory colonies were unsuccessful. Therefore, we developed a simple bioassay adjusted for soil-feeding termites, which we applied to freshly collected field colonies. Female nymphs from non-dispersing colonies proved to be very responsive to orphaning, which allowed successful testing of RNERO effects. This experimental design should facilitate functional tests of the queen- and egg-specific volatiles identified in other South American Syntermitinae, i.e., (6*E*)-nerolidol in *Silvestritermes heyeri*, *Cyrilliotermes angulariceps* and *Labiotermes labralis*, and (5*Z*,9*S*)-tetradec-5-en-9-olide in *S. minutus*, the former three having colonies almost exclusively headed by primaries, and the last being an emblematic case of AQS^22^. The potential function of these volatiles is even more interesting when considering the different chemical nature and presumed biosynthesis of the sesquiterpene nerolidol and the macrolide tetradecenolide, likely arising through cyclization of hydroxy fatty acid. Yet a different biosynthetic origin can be expected for *n*-butyl-*n*-butyrate and 2-methylbutan-1-ol in *R. speratus*, suggesting altogether multiple independent co-options of fundamentally different secondary metabolites for queen pheromone function in different termite taxa. This chemical diversity of termite QPPs (or presumed QPPs) is unexpected given their vital importance for the social homeostasis in termite colonies and contrasts with the conservative situation in eusocial Hymenoptera (except for the honeybee), in which structurally related CHCs have been recruited for the QPP function across the multiple independently arising eusocial lineages.

In contrast to *R. speratus*, in which the volatile QPP also has a releaser function by attracting workers^15^, we did not observe any influence of RNERO on worker or soldier behavior, even though the compound has electroantennographic activity also in workers^39^. Hence, it is likely that other compounds may be responsible for physical queen recognition. Recent observations in the genus *Reticulitermes* have shown that recognition of reproductives, accompanied by appropriate behavior, is mediated by king/queen-specific CHCs^29,30,45^. Therefore, in line with some predictions (e.g. ref. ^46^) and reports of differential CHC profiles in reproductive and sterile colony members^27,28^, termite fertility signaling may consist of multiple components, such as volatile queen (king) primer pheromones suppressing the reproductive potential of nestmates and contact releaser pheromone cues contained in the CHC profiles.

The apparent correlation between RNERO production and queen fertility, as well as RNERO presence on eggs, complies with the definition of an honest signal, truly reflecting queen fertility^4,6,14,47^. It would be of interest to study whether RNERO production is subject to the same endocrine regulation as fertility itself, i.e., intuitively the juvenile hormone pathway, as evidenced for sex/aggregation volatile pheromones in solitary insects (e.g. refs. ^48–50^), but also for fertility-linked CHC profiles of termites^51^ and eusocial Hymenoptera, including those confirmed as queen pheromones^14,52^. Enantioselective biosynthesis of RNERO, presumably from farnesyl diphosphate precursor, would be another appealing topic of investigation, given the recent advances in functional characterizations of terpene synthases in insects, which revealed to be unrelated to the terpene synthases known in plants and appear to have evolved independently in individual insect lineages from isoprenyl diphosphate synthases^53–56^. Along with the biosynthesis, the production organ of the queen pheromone should also be located, because it has not been identified in either *E. neotenicus* or *R. speratus*^15,39^. Last but not least, the role of RNERO should be addressed from the perspective of its possible ancestral function, preceding its co-option for the queen pheromone signal. Such an evolutionary pattern has been proposed for the queen pheromone of *R. speratus* and may also be realistic for RNERO, given the wide range of reported biological functions of different nerolidol isomers, including antimicrobial properties^57^.

## Methods

### Origin of termites, sampling

Ten colonies (A–J) of *Embiratermes neotenicus* (Holmgren 1906) were used in this study. The colonies were collected in French Guiana in February 2019, March 2020, and February 2022 at three forest localities along the Road to Petit Saut (N5°04.250′ W52°58.770′–N5°04.650′ W53°01.360′) and one locality 13 km South-West from Kourou (N5°06.440′ W52°45.521′).

*E. neotenicus* colonies live in soil-made nests situated at the ground level of the forest, whose volume may reach up to multiple dozen liters. We collected large nests, having more than 50 l in volume. In such colonies, the founding queen has already been replaced by multiple NQs, and they contain, throughout the year, tens to hundreds of parthenogenetic NY4**♀**. Prior to dispersal flights which take place in July^23,58^, most mature colonies also produce up to thousands of male and female nymphs of sexual origin. Thus, the groups of parthenogenetic NY4**♀** are for some time (January–June) diluted in the pool of future dispersers. However, not all large (and supposedly mature) colonies produce dispersers every year. This offered us the opportunity to collect colonies preparing for dispersal via hundreds of nymphs of both sexes as well as non-dispersing colonies containing tens to hundreds of supposedly parthenogenetic NY4**♀** and only a few individual male nymphs.

Entire nests were kept intact in large plastic jars protected from direct sunlight in the Laboratory Hydreco (Petit Saut Dam, French Guiana) for a maximum of 2 days. The colonies were dissected immediately prior to the experiment, and the specimens needed for bioassays and for genetic and/or chemical analyses were collected. Fresh individuals were collected from previously undisturbed parts of the nest for the daily replacement of workers and soldiers in the bioassays.

Individual castes can be easily recognized based on their external anatomy. Different nymphal stages can be distinguished based on the head size, shape, and length of wing pads. Freshly molted NQs are non-pigmented (white) and non-physogastric, but can easily be separated from NY4 based on the short wing rudiments and larger compound eyes (Fig. 2). Sex of nymphs and non-physogastric neotenics was recognized under stereomicroscope based on the shapes of abdominal sternites, especially the enlarged sternite 6 in females.

### Chemical analyses

In the three colonies used for queen inhibition bioassays (G–I), we verified the presence of RNERO in mature NQs and other available castes. Individuals were extracted in clean glass vials using 30 µl of *n*-hexane (GC-MS grade, Merck) per individual (or per approx. 50 eggs) for 10 min at room temperature. Extracts were transferred into clean vials and stored at −20 °C. We used two-dimensional gas chromatography coupled with mass spectrometry (GC×GC-TOFMS, Pegasus 4D Leco, St. Joseph, MI, USA), equipped with a combination of non-polar ZB-5MS (30 m, internal diameter 0.25 mm, film thickness 0.25 μm, Phenomenex, Torrance, CA, USA) and medium polarity BPX-50 (1.3 m, internal diameter 0.1 mm, film thickness 0.1 μm, Restek, Bellefonte, PA, USA) columns. The temperature program for the primary column was 50 °C (1 min) to 320 °C (20 min) at 8 °C/min; the secondary column was set 10 °C higher. We identified RNERO based on Kovats retention index (1566), elution time comparison with synthetic standard and EI-MS fragmentation pattern^39^. Relative RNERO abundance was compared by simultaneous visualization of all chromatograms from a given colony on an identical scale of detector intensities in Leco ChromaTOF software.

### Origin of chemicals

(3*S*,6*E*)-nerolidol, (*E*,*E*)-α-farnesene, and (*E*,*E*)-farnesol were purchased from Merck (Darmstadt, Germany). RNERO was obtained from our previous synthesis and enantiomeric purity of (3*S*,6*E*)-nerolidol and RNERO were established by chiral GC^39^.

### Statistics and reproducibility

The results presented here originate from the data on 10 different field colonies. For Experiment I, six colonies was used and each of them was treated as one replicate in a comparison of three dispersing vs. three non-dispersing colonies. For Experiments II and III, each colony was considered as an independent unit and statistically evaluated separately from other colonies, with five replicates per each treatment within each colony: replicate groups originated through random sampling of individuals belonging to appropriate castes during the dissection of the colonies. Each individual was only used in one single replicate of one single experiment.

To compare the cumulative numbers of new queens in Experiments I–III across the five replicates of different treatments, survival curves were constructed and compared using Mantel–Cox Test, with subsequent pairwise comparisons among treatments being performed using Mantel–Cox Test with Sidak-corrected 0.05 *α* value. Numbers of new NQs in individual replicate groups of each treatment were compared by means of non-parametric tests, i.e. Kruskal–Wallis rank-sum test and subsequent pairwise comparisons using Dunn’s test.

In electroantennographic measurements, non-dispersing colony J was used and tested NY4♀ randomly selected. Each nymph was only used for one stimulation series. Parametric analysis of variance was used (ANOVA) and the data was appropriately transformed prior to the analysis to comply with the assumptions of parametric testing.

Details on statistical evaluation are given below together with the design of individual experiments. All calculations were performed and graphs were generated in GraphPad Prism v 8.0 (GraphPad Software Inc., San Diego, CA, USA).

### Bioassays

Bioassays were conducted in Laboratory Hydreco at 28 °C, elevated humidity and permanent darkness, dim laboratory light was used during the regular census of experimental groups.

#### Experiment I—readiness of NY4♀ to molt into NQs

The design of the experiment is summarized in Fig. 2a. We established six groups of 17 NY4**♀**, originating from six different colonies (A–F). Three of these colonies (A–C) were considered non-dispersing since they contained only a few tens of NY4 (51–168) and a low proportion of male NY4 (2–5%). The other three colonies (D–F) were apparently nearing dispersal and contained numerous male and female nymphs (stages 2–4). Most of these nymphs were of stage NY4 (from 667 to more than 1200) and the proportion of males among NY4 ranged from 41% to 42%.

NY4**♀** were placed in plastic Petri dishes (6 cm in diameter) lined with a Whatman No. 1 Grade filter paper moistened with 0.2 ml distilled water. The groups were controlled every 6 h and newly differentiated NQs were scored and removed. Every 12 h, 0.1 ml water was added. The experiment was terminated after 72 h. Total cumulative counts of newly differentiated NQs from non-dispersing vs. dispersing colonies were compared across the timepoints using Mantel–Cox Test.

#### Experiments II and III—queen inhibition bioassays

Queen inhibition bioassays were performed with three different mature colonies. Two of them, colonies G and H, were typical non-dispersing colonies with 269 and 249 NY4**♀**, respectively, and a low proportion of males (1.5% and 1%). The third colony (I) contained a low number of NY4 (261), but a relatively elevated proportion of males (32%), suggesting that besides parthenogenetic NY4**♀** also a non-negligible number of sexually produced females was present in the colony.

To establish the genetic origin of NY4**♀** and new NQs in the three colonies, a subset of NY4**♀** and all NQs that developed in the experiments were genotyped as described below.

In the initial experiment II with colony G, we tested (i) whether the presence of 10 mature NQs inhibits the formation of new NQs in groups of NY4**♀**, (ii) whether the amount of RNERO equivalent to headspace emission of 10 NQs mimics the effect of living NQs and inhibits the formation of new NQs, and (iii) whether 1 RNERO equivalent will also suppress NQ differentiation. The bioassay design is summarized in Fig. 4a. Groups of termites were established in new 9 cm plastic Petri dishes lined with filter paper moistened with 0.7 ml distilled water. Each dish contained 9 NY4**♀**, 20 workers, and 5 soldiers. To prevent mortality and ensure a fresh workforce allowing trophallactic feeding of dependent castes (soldiers, nymphs, queens), the workers and soldiers were replaced with new individuals (freshly collected from an undisturbed part of the nest) every 24 h. Every 6 h, the census of all individuals in the groups was performed and newly molted NQs were removed. Every 12 h, the chemical treatments were renewed, and 0.2 ml of water was added. The bioassay was terminated after 72 h. Termite groups were subjected to four treatments (control, 10NQ, 1QE, and 10QE), always with five replicates. Chemical treatments were applied as 10 µl of *n*-hexane solution on an 8 × 8 mm piece of filter paper, placed in the middle of the dish, allowing both evaporation of the substances to the headspace of the dish and direct contact of the termites with the paper. In control groups, the filter paper was treated with hexane only. In 10NQ treatment, 10 mature NQs from the same colony were added to the group together with the hexane-treated paper. In 1QE and 10QE treatments, the paper was treated with 1 or 10 equivalents, respectively, of the headspace emissions of RNERO by one mature NQ per 12 h, i.e. 260 or 2600 ng^39^.

The design of the subsequent experiment III with colonies H and I are summarized in Fig. 5a. In this experiment, we tested whether the 10QE treatment, which revealed to suppress the NQ differentiation in colony G, will be effective as an airborne signal, without direct access of the termites to its source. Therefore, the bioassay used three treatments, i.e. control, 10NQ and 10QE#; in the latter treatment, the treated filter paper was surrounded by a fine double mesh (2 cm outer diameter) made of copper wire, preventing the termites from antennal contact with the paper. Experiment III was run for 60 h. All other parameters were identical to experiment II.

The counts of newly differentiated NQs in all queen inhibition experiments were addressed from two perspectives and separately for each colony. First, we considered the cumulative counts of new NQs across replicates of individual treatments at each datapoint (every 6 h). From these counts, we constructed the survival curves with new NQs being treated as subjects experiencing an event and the non-differentiated NY4**♀** as censored subjects. The survival curves were compared among all treatments using Mantel-Cox Test, and then pairwise comparisons of the survival curves for individual pairs of treatments were performed using Mantel–Cox Test with Sidak-corrected 0.05 *α* value. Second, the numbers of new NQs in individual replicate groups of each treatment were compared at the end of the experiment using the Kruskal–Wallis rank-sum test, followed by Dunn’s multiple comparisons among pairs of treatments.

### Microsatellite genotyping

To assess the genetic origin (sexual or parthenogenetic) of NY4**♀** and NQs in the inhibition bioassays (colonies G–I), we genotyped a subset of NY4**♀** and all NQs from the bioassays, together with workers and soldiers. The specimens were sampled into 96% ethanol and genotyped at 9 polymorphic microsatellite loci developed in our previous studies^23,44^. Primers are listed and methods are described in detail in Supplementary Methods and Supplementary Table 2.

We successfully genotyped 12 workers, 12 soldiers, 38 NY4**♀**, and 23 NQs from colony G, 13 workers, 12 soldiers, 44 NY4**♀**, and 10 NQs from colony H, and 14 workers, 9 soldiers, 65 NY4**♀** and 7 new NQs from colony I. From allelic combinations in sterile castes, we inferred the genotypes of the putative pair of founding primaries. Subsequently, we were able to distinguish NY4**♀** and NQs of sexual origin, carrying exclusive alleles of both parents at all loci at which they would be expected, from those that were missing the exclusive alleles of one of the parents (intuitively of the primary king) and were thus of parthenogenetic origin.

### Electrophysiology

Central part of colony J, containing NQs, a primary king, a cohort of NY4**♀**, workers and soldiers, was transported to the laboratory in Prague. According to the relatively low number of NY4**♀** and low male proportion among NY4**♀**, the colony was considered a non-dispersing colony. The nymphs were kept in the colony fragment with queens and sterile castes until the experiment to prevent their differentiation into NQs.

Antennal responses of NY4**♀** were measured for RNERO, it’s opposite (*S*) enantiomer, and two structurally related sesquiterpenoids, (*E*,*E*)-α-farnesene and (*E*,*E*)-farnesol. Opened last flagellomere and brain were fixed between two Ag/AgCl electrodes containing Ringer’s solution and connected to a high impedance (10^14^ Ω) amplifier (Syntech, Buchenbach, Germany). The antennal preparation was placed into a stream of cleaned air, into which stimuli were injected from Pasteur pipettes with a 1.5 cm^2^ filter paper impregnated with 10 µl of the test solution. Odor injections were controlled by a foot switch-operated Syntech stimulus controller and maximal negative deflection was quantified using Syntech EAG software.

Two independent experiments were performed, one with 50 ng and the other with 500 ng of the four compounds in *n*-hexane. In each experiment, 15 antennae of 15 NY4**♀** were stimulated with a series of the four stimuli in randomized order, preceded and followed with hexane-treated and non-treated papers as controls. New Pasteur pipettes were prepared for each series, and the data from each series was related to mean responses to hexane stimulations. The datasets were log_2_-transformed to reduce heteroscedasticity and comply with assumptions of variance equality for parametric testing (Brown-Forsythe test and Bartlett test for equal variances, and Shapiro-Wilk normality test) and then compared among treatments using ANOVA followed by Tukey post-hoc test.

### Reporting summary

Further information on research design is available in the Nature Research Reporting Summary linked to this article.

### Supplementary information

Supplementary Information

Description of Additional Supplementary Files

Supplementary Data 1

Reporting Summary file

## Data Availability

Data generated or analyzed during this study are included in Supplementary Data 1 file and in Supplementary information file. Supplementary information file contains the gas chromatography–mass spectrometry data, the genotypes of the 259 genotyped individuals, detailed methods, characteristics of 9 microsatellite markers and corresponding primers.
