# Author Correction: Identification of a queen primer pheromone in higher termites

**DOI:** 10.1038/s42003-024-06150-4

**Published:** 2024-04-16

**Authors:** Klára Dolejšová, Jan Křivánek, Jitka Štáfková, Natan Horáček, Jana Havlíčková, Virginie Roy, Blanka Kalinová, Amit Roy, Pavlína Kyjaková, Robert Hanus

**Affiliations:** 1 Chemistry of Social Insects, Institute of Organic Chemistry and Biochemistry of the Czech Academy of Sciences, Prague, Czech Republic; 2 Faculty of Science, Charles University, Prague, Czech Republic; 3 Present Address: Université Paris Est Créteil, Sorbonne Université, Université Paris Cité, CNRS, INRAE, IRD, iEES Paris, Créteil, France; 4 Czech University of Life Sciences, Prague, Czech Republic

**Keywords:** Animal physiology, Entomology

Correction to: *Communications Biology* 10.1038/s42003-022-04163-5, published online 02 November 2022

In the original version of the Article, the Acknowledgements contained an error in funding sources, stating “The Grant Agency of the Charles University in Prague (580413) (to NH)”. The text should read “Charles University Grant Agency (project GA UK No 371021, N.H.)”. The original article has been corrected.

