# Supplementary material for: Identification of a queen primer pheromone in higher termites
Source: Commun Biol. 2022 Nov 2;5:1165. doi: 10.1038/s42003-022-04163-5 (PMC9630296; doi:10.1038/s42003-022-04163-5)
Supplement: Supplementary file 1 — Supplementary Information [file 42003_2022_4163_MOESM1_ESM.pdf]

Identification of a queen primer pheromone in higher termites

SUPPLEMENTARY INFORMATION

Klára Dolejšová[1,2,†], Jan Křivánek[1,†], Jitka Štáfková[1], Natan Horáček[1,2], Jana Havlíčková[1], Virginie Roy[3], Blanka Kalinová[4], Amit Roy[4], Pavlína Kyjaková[1] & Robert Hanus[1,*]

[1] Chemistry of Social Insects, Institute of Organic Chemistry and Biochemistry of the Czech Academy of Sciences, Prague, Czech Republic

[2] Faculty of Science, Charles University, Prague, Czech Republic

[3] Université Paris Est Créteil, Sorbonne Université, CNRS, INRAE, IRD, iEES Paris, Créteil, France

[4] Czech University of Life Sciences, Prague, Czech Republic

SUPPLEMENTARY METHODS

gDNA extraction, PCR, genotyping

Termite heads were placed in wells of a 96-well PCR plate. START-Blue reagent from the DEP-25 DNA Extraction Kit (Top-Bio, Vestec, Czech Republic) was added at 25 µl per well. The capped plate was vortexed for 30 seconds and centrifuged (5 minutes, 200 g). The wells were inspected to check the contact of termite samples with the reagent. The plates were incubated at 95°C for 20 minutes and after cooling down, 25 µl STOP solution per well was added. Reagents were mixed by inverting the plate. The resulting liquid sample was used as template in PCR.

Individuals were genotyped for nine microsatellite loci[1,2]. PCRs were performed in a total volume of 6µL containing 1X Qiagen Multiplex PCR Master Mix, 0.2 µM of each forward and reverse primer, 1 µL template DNA and PCR-grade water (q.s.). Sequences of primers are indicated in Table S2 below. PCR conditions were an initial denaturation step at 95 °C for 5 min, 35 amplification cycles at 95 °C for 30 s, 60 °C for 90 s, 72 °C for 30 s, and a final extension at 68 °C for 10 min. Genotyping was performed using a SeqStudio Genetic Analyzer (Applied Biosystems, genomic platform of IMRB, Mondor Institute, France). Fragment lengths were manually evaluated on chromatograms to detect inconsistencies, and genotypes were scored against the GeneScan-500 Liz Size Standard (Applied Biosystems) using GeneMapper 5 (Applied Biosystems).

SUPPLEMENTARY FIGURES AND TABLES

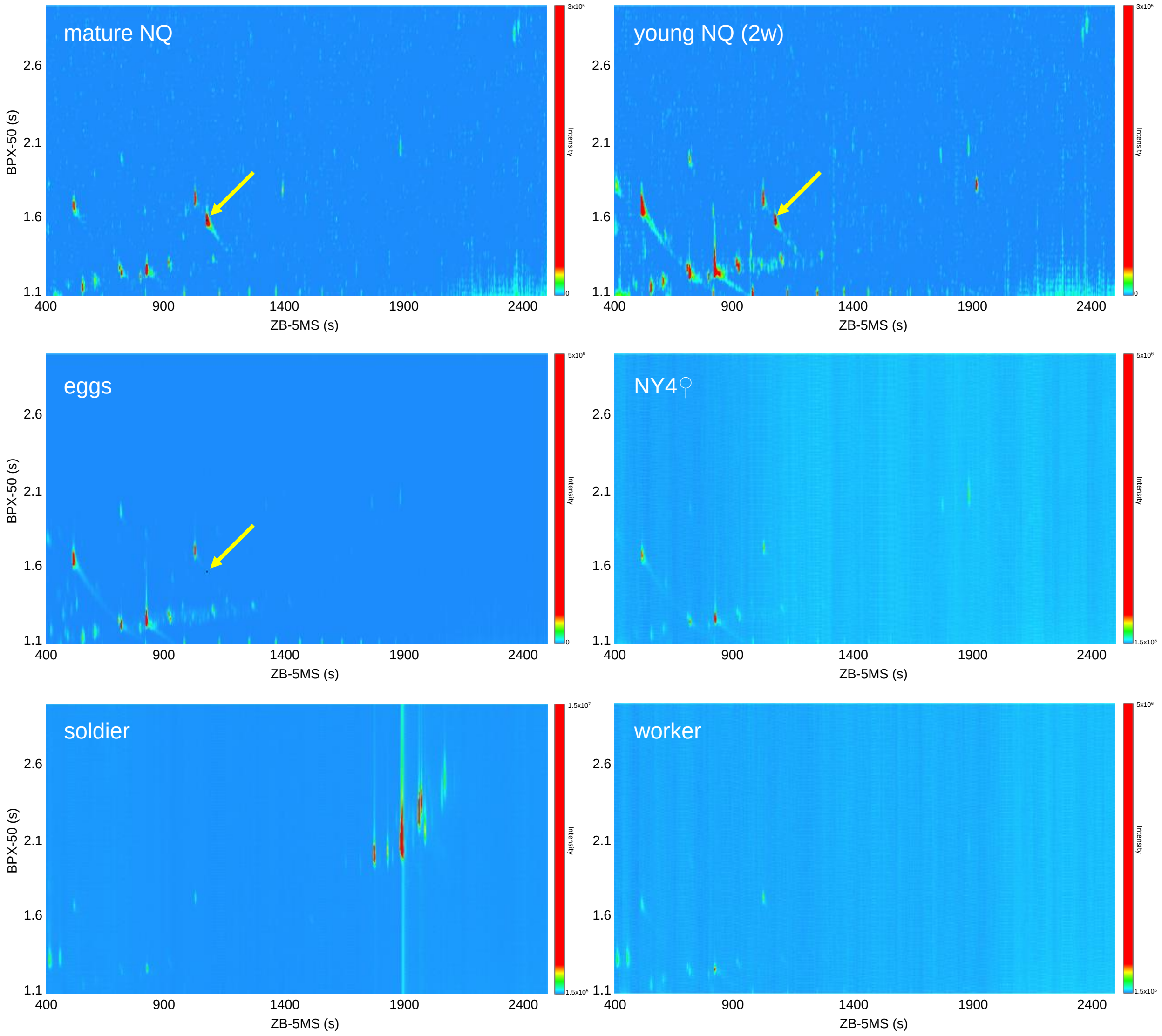

Supplementary Figure 1. GC×GC chromatograms of body washes of different castes and life stages from colony G (related to Figure 3). The peak of RNERO is indicated with an arrow. See Fig. S4 for MS spectra comparison.

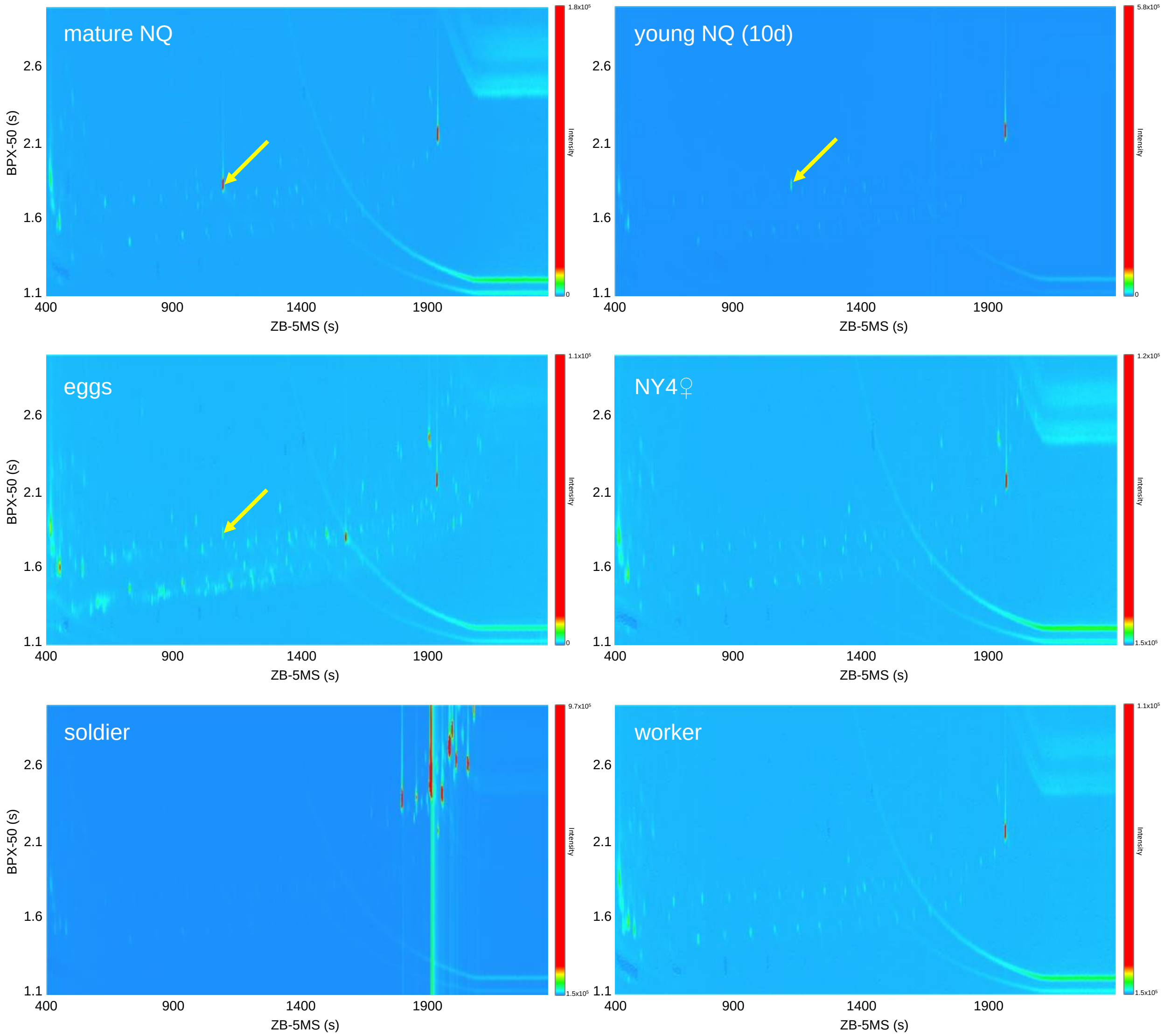

Supplementary Figure 2. GC×GC chromatograms of body washes of different castes and life stages from colony H (related to Figure 3). The peak of RNERO is indicated with an arrow. See Fig. S4 for MS spectra comparison.

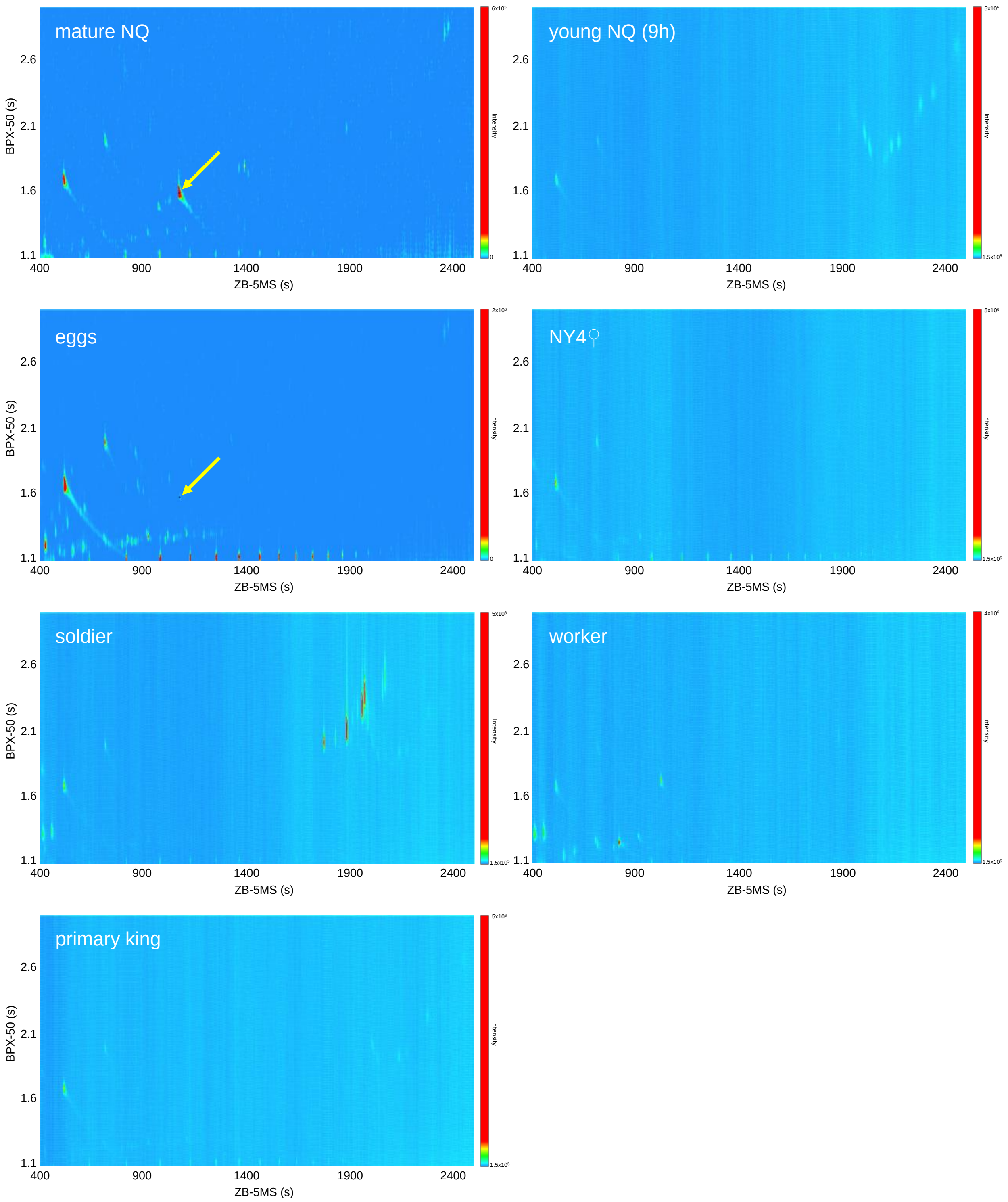

Supplementary Figure 3. GC×GC chromatograms of body washes of different castes and life stages from colony I, related to Figure 3. The peak of RNERO is indicated with an arrow. See Fig. S4 for MS spectra comparison.

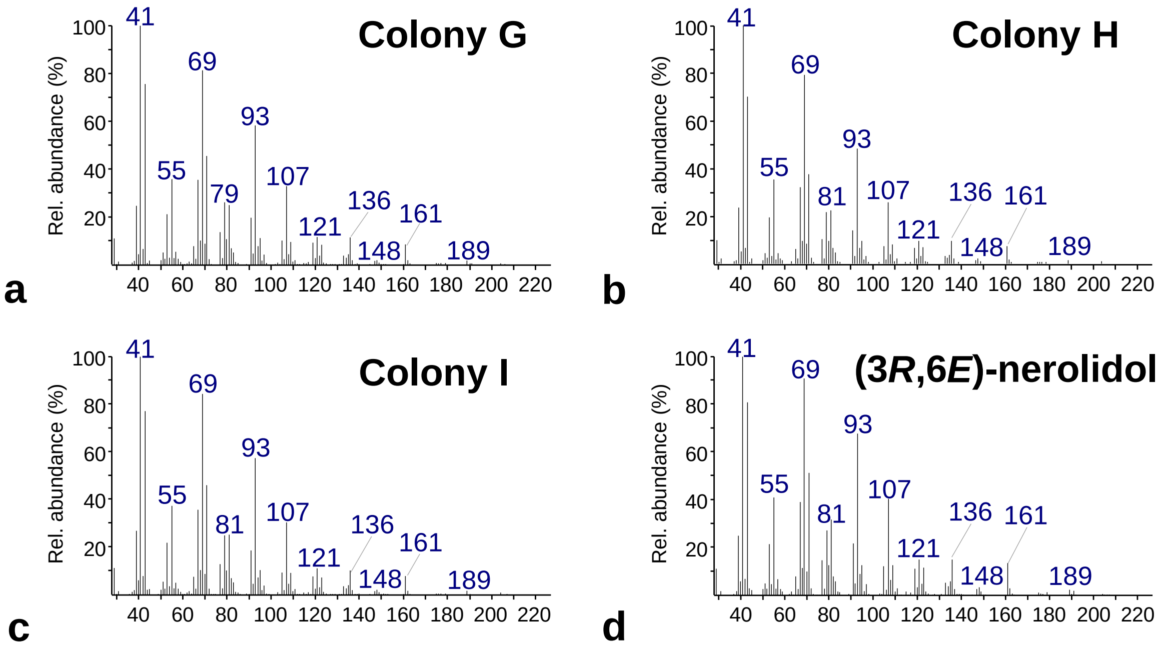

Supplementary Figure 4. Electron ionization mass spectra of RNERO detected in the body washes of mature NQs from colonies G, H and I (a, b, c) and of synthetic (3*R*,6*E*)-nerolidol (d) (related to Figure 3).

Supplementary Table 1. Genotypes recorded in colonies G, H and I. In red bold exclusive paternal alleles, in blue italics genotypes inconsistent with sexual origin of nymphs and neotenic queens (related to Figures 4 and 5).

COLONY G

Caste<br>n		En-8	En-10	En-11	En-15	En-19	En-25	En-35	En-37	En-39	genetic origin
**primary queen** *(inferred)*		157 157	103 113	162 162	286 286	178 190	87 95	119 119	135 147	158 158	
**primary king** *(inferred)*		157 157	103 103	**166 166**	**298 284**	**192 192**	95 87	119 119	**131 131**	158 158	
**workers**<br>12		157 157 *(12)*	103 103 *(5)*	162 **166** *(12)*	**284** 286 *(4)*	178 **192** *(5)*	87 87 *(3)*	119 119 *(12)*	**131** 135 *(5)*	158 158 *(12)*	
			103 113 *(7)*		286 **298** *(8)*	190 **192** *(7)*	87 95 *(5)*		**131** 147 *(7)*		
							95 95 *(4)*				
**soldiers**<br>12		157 157 *(12)*	103 103 *(6)*	162 **166** *(12)*	**284** 286 *(6)*	178 **192** *(6)*	87 87 *(3)*	119 119 *(12)*	**131** 135 *(4)*	158 158 *(12)*	
			103 113 *(6)*		286 **298** *(6)*	190 **192** *(6)*	87 95 *(5)*		**131** 147 *(8)*		
							95 95 *(4)*				
**nymphs (NY4♀)**<br>38	1	157 157	103 113	*162 162*	*286 286*	*178 190*	95 95	119 119	*135 147*	158 158	*parthenogenesis*
	2	157 157	113 113	*162 162*	*286 286*	*190 190*	87 87	119 119	*147 147*	158 158	*parthenogenesis*
	3	157 157	103 113	*162 162*	*286 286*	*178 190*	95 95	119 119	*135 147*	158 158	*parthenogenesis*
	4	157 157	103 103	*162 162*	**284** 286	178 **192**	87 95	119 119	*135 147*	158 158	*parthenogenesis*
	5	157 157	103 113	*162 162*	*286 286*	*190 190*	95 95	119 119	*135 147*	158 158	*parthenogenesis*
	6	157 157	113 113	*162 162*	**284** 286	*190 190*	87 95	119 119	*135 147*	158 158	*parthenogenesis*
	7	157 157	103 113	*162 162*	*286 286*	*178 190*	95 95	119 119	*135 147*	158 158	*parthenogenesis*
	8	157 157	103 113	*162 162*	*286 286*	*178 190*	87 95	119 119	*135 147*	158 158	*parthenogenesis*
	9	157 157	103 113	*162 162*	*286 286*	*178 190*	95 95	119 119	*135 147*	158 158	*parthenogenesis*
	10	157 157	103 113	*162 162*	*286 286*	*178 190*	87 95	119 119	*135 147*	158 158	*parthenogenesis*
	11	157 157	113 113	*162 162*	*286 286*	*190 190*	95 95	119 119	*147 147*	158 158	*parthenogenesis*
	12	157 157	103 113	*162 162*	*286 286*	*190 190*	95 95	119 119	*135 147*	158 158	*parthenogenesis*
	13	157 157	103 113	*162 162*	*286 286*	*178 190*	87 87	119 119	*135 147*	158 158	*parthenogenesis*
	14	157 157	103 113	*162 162*	*286 286*	*178 190*	87 87	119 119	*135 147*	158 158	*parthenogenesis*
	15	157 157	103 113	*162 162*	*286 286*	*178 190*	95 95	119 119	*135 147*	158 158	*parthenogenesis*
	16	157 157	103 103	*162 162*	*286 286*	178 **192**	87 87	119 119	*135 147*	158 158	*parthenogenesis*
	17	157 157	113 113	*162 162*	*286 286*	*190 190*	87 95	119 119	*135 147*	158 158	*parthenogenesis*
	18	157 157	103 113	*162 162*	*286 286*	190 **192**	87 95	119 119	*135 147*	158 158	*parthenogenesis*
	19	157 157	103 113	*162 162*	**284** 286	190 **192**	87 95	119 119	*135 147*	158 158	*parthenogenesis*
	20	157 157	103 113	*162 162*	*286 286*	190 **192**	87 95	119 119	*135 147*	158 158	*parthenogenesis*
	21	157 157	113 113	*162 162*	**284** 286	*190 190*	87 95	119 119	*135 147*	158 158	*parthenogenesis*
	22	157 157	103 113	*162 162*	*286 286*	*178 190*	87 95	119 119	*135 147*	158 158	*parthenogenesis*
	23	157 157	113 113	*162 162*	*286 286*	*178 178*	87 95	119 119	*135 147*	158 158	*parthenogenesis*
	24	157 157	103 103	*162 162*	*286 286*	*178 178*	87 87	119 119	*147 147*	158 158	*parthenogenesis*
	25	157 157	113 113	*162 162*	**284** 286	*190 190*	87 95	119 119	*135 147*	158 158	*parthenogenesis*
	26	157 157	103 103	*162 162*	**284** 286	**192 192**	87 87	119 119	*135 147*	158 158	*parthenogenesis*
	27	157 157	103 113	*162 162*	*286 286*	*178 190*	87 87	119 119	*135 147*	158 158	*parthenogenesis*
	28	157 157	113 113	*162 162*	*286 286*	*190 190*	95 95	119 119	*147 147*	158 158	*parthenogenesis*
	29	157 157	103 113	*162 162*	*286 286*	*178 190*	95 95	119 119	*135 147*	158 158	*parthenogenesis*
	30	157 157	103 113	*162 162*	*286 286*	*178 190*	87 95	119 119	*135 147*	158 158	*parthenogenesis*
	31	157 157	103 113	*162 162*	**284** 286	*178 190*	87 95	119 119	*135 147*	158 158	*parthenogenesis*
	32	157 157	103 113	*162 162*	*286 286*	*178 190*	87 87	119 119	*135 147*	158 158	*parthenogenesis*
	33	157 157	103 103	*162 162*	*286 286*	*178 178*	87 87	119 119	*147 147*	158 158	*parthenogenesis*
	34	157 157	103 113	*162 162*	*286 286*	*178 190*	87 95	119 119	*135 147*	158 158	*parthenogenesis*
	35	157 157	103 103	*162 162*	*286 286*	*190 190*	87 95	119 119	*135 147*	158 158	*parthenogenesis*
	36	157 157	103 113	*162 162*	*286 286*	*178 190*	87 95	119 119	*135 135*	158 158	*parthenogenesis*
	37	157 157	103 113	*162 162*	**284** 286	190 **192**	87 87	119 119	*135 135*	158 158	*parthenogenesis*
	38	157 157	103 113	*162 162*	*286 286*	*178 190*	87 95	119 119	*135 147*	158 158	*parthenogenesis*
**new neotenic queens**<br>23	1	157 157	103 113	162 **166**	286 **298**	190 **192**	87 95	119 119	**131** 147	158 158	*sex*
	2	157 157	103 113	*162 162*	*286 286*	*178 190*	87 95	119 119	*135 147*	158 158	*parthenogenesis*
	3	157 157	103 113	*162 162*	*286 286*	*178 190*	87 95	119 119	*135 147*	158 158	*parthenogenesis*
	4	157 157	103 113	*162 162*	*286 286*	*178 190*	87 95	119 119	*135 147*	158 158	*parthenogenesis*
	5	157 157	103 113	*162 162*	*286 286*	*178 190*	87 87	119 119	*135 147*	158 158	*parthenogenesis*
	6	157 157	103 113	*162 162*	*286 286*	*178 190*	87 95	119 119	*135 147*	158 158	*parthenogenesis*
	7	157 157	103 113	*162 162*	*286 286*	*178 190*	95 95	119 119	*135 147*	158 158	*parthenogenesis*
	8	157 157	103 103	*162 162*	*286 286*	*178 178*	95 95	119 119	*147 147*	158 158	*parthenogenesis*
	9	157 157	103 113	*162 162*	*286 286*	*178 190*	87 95	119 119	*135 147*	158 158	*parthenogenesis*
	10	157 157	103 113	*162 162*	*286 286*	*190 190*	87 95	119 119	*135 135*	158 158	*parthenogenesis*
	11	157 157	103 113	*162 162*	**284** 286	*190 190*	87 95	119 119	*135 135*	158 158	*parthenogenesis*
	12	157 157	103 113	*162 162*	*286 286*	*178 190*	87 95	119 119	*135 147*	158 158	*parthenogenesis*
	13	157 157	103 103	*162 162*	**284** 286	178 **192**	87 95	119 119	*135 135*	158 158	*parthenogenesis*
	14	157 157	103 113	*162 162*	*286 286*	*178 190*	87 95	119 119	*135 147*	158 158	*parthenogenesis*
	15	157 157	103 113	*162 162*	*286 286*	*178 190*	87 95	119 119	*135 147*	158 158	*parthenogenesis*
	16	157 157	103 113	*162 162*	**284** 286	*178 190*	87 95	119 119	*135 147*	158 158	*parthenogenesis*
	17	157 157	113 113	*162 162*	*286 286*	*190 190*	95 95	119 119	*135 147*	158 158	*parthenogenesis*
	18	157 157	103 113	*162 162*	*286 286*	*190 190*	95 95	119 119	*135 147*	158 158	*parthenogenesis*
	19	157 157	103 113	*162 162*	**284** 286	*178 190*	87 87	119 119	*135 147*	158 158	*parthenogenesis*
	20	157 157	103 113	*162 162*	*286 286*	*178 190*	95 95	119 119	*135 147*	158 158	*parthenogenesis*
	21	157 157	103 113	*162 162*	*286 286*	*190 190*	95 95	119 119	*135 147*	158 158	*parthenogenesis*
	22	157 157	103 113	*162 162*	*286 286*	*178 190*	87 95	119 119	*135 147*	158 158	*parthenogenesis*
	23	157 157	103 113	*162 162*	**284** 286	190 **192**	87 87	119 119	*135 147*	158 158	*parthenogenesis*

COLONY H

Caste<br>n		En-8	En-10	En-11	En-15	En-19	En-25	En-35	En-37	En-39*	genetic origin
**primary queen** *(inferred)*		162 162	103 115	157 157	192 192	284 286	89 97	121 121	135 135	- -	
**primary king** *(inferred)*		162 162	**111** **113**	157 **167**	192 192	284 286	89 89	121 121	135 135		
**workers**<br>13		162 162 *(13)*	103 **113** *(4)*<br>103 **111** *(2)*<br>**113** 115 *(5)*<br>**111** 115 *(2)*	157 157 *(8)*<br>157 **167** *(5)*	192 192 *(13)*	284 284 *(5)*<br>284 286 *(6)*<br>286 286 *(2)*	89 89 *(7)*<br>89 97 *(6)*	121 121 *(13)*	135 135 *(13)*	- -	
**soldiers**<br>12		162 162 *(12)*	103 113 *(5)*<br>103 **111** *(4)*<br>113 115 *(1)*<br>**111** 115 *(2)*	157 157 *(6)*<br>157 **167** *(6)*	192 192 *(12)*	284 284 *(1)*<br>284 286 *(3)*<br>286 286 *(8)*	89 89 *(6)*<br>89 97 *(6)*	121 121 *(12)*	131 135 *(12)*	- -	
**nymphs (NY4♀)**<br>44	1	162 162	*103 115*	157 157	192 192	284 286	89 89	121 121	135 135	- -	*parthenogenesis*
	2	162 162	*103 115*	157 157	192 192	284 286	89 97	121 121	135 135	- -	*parthenogenesis*
	3	162 162	*103 115*	157 157	192 192	284 286	89 97	121 121	135 135	- -	*parthenogenesis*
	4	162 162	*103 115*	157 157	192 192	284 286	*97 97*	121 121	135 135	- -	*parthenogenesis*
	5	162 162	*103 115*	157 157	192 192	284 286	89 97	121 121	135 135	- -	*parthenogenesis*
	6	162 162	*103 115*	157 157	192 192	284 286	89 97	121 121	135 135	- -	*parthenogenesis*
	7	162 162	*103 115*	157 157	192 192	286 286	89 89	121 121	135 135	- -	*parthenogenesis*
	8	162 162	*103 115*	157 157	192 192	284 286	89 97	121 121	135 135	- -	*parthenogenesis*
	9	162 162	*103 115*	157 157	192 192	286 286	89 97	121 121	135 135	- -	*parthenogenesis*
	10	162 162	*103 115*	157 157	192 192	286 286	89 89	121 121	135 135	- -	*parthenogenesis*
	11	162 162	*103 115*	157 157	192 192	284 286	89 97	121 121	135 135	- -	*parthenogenesis*
	12	162 162	*103 115*	157 157	192 192	284 284	*97 97*	121 121	135 135	- -	*parthenogenesis*
	13	162 162	*103 115*	157 157	192 192	284 286	*97 97*	121 121	135 135	- -	*parthenogenesis*
	14	162 162	*103 115*	157 157	192 192	284 286	89 89	121 121	135 135	- -	*parthenogenesis*
	15	162 162	*103 115*	157 157	192 192	286 286	89 97	121 121	135 135	- -	*parthenogenesis*
	16	162 162	*103 115*	157 157	192 192	284 286	89 89	121 121	135 135	- -	*parthenogenesis*
	17	162 162	*103 115*	157 157	192 192	286 286	89 97	121 121	135 135	- -	*parthenogenesis*
	18	162 162	*103 115*	157 157	192 192	284 286	89 97	121 121	135 135	- -	*parthenogenesis*
	19	162 162	*103 115*	157 157	192 192	286 286	89 97	121 121	135 135	- -	*parthenogenesis*
	20	162 162	*103 115*	157 157	192 192	286 286	89 97	121 121	135 135	- -	*parthenogenesis*
	21	162 162	*103 115*	157 157	192 192	286 286	89 97	121 121	135 135	- -	*parthenogenesis*
	22	162 162	*103 115*	157 157	192 192	284 286	89 97	121 121	135 135	- -	*parthenogenesis*
	23	162 162	*103 115*	157 157	192 192	284 284	*97 97*	121 121	135 135	- -	*parthenogenesis*
	24	162 162	*103 115*	157 157	192 192	284 286	89 97	121 121	135 135	- -	*parthenogenesis*
	25	162 162	*103 115*	157 157	192 192	286 286	89 97	121 121	135 135	- -	*parthenogenesis*
	26	162 162	*103 115*	157 157	192 192	284 286	89 97	121 121	135 135	- -	*parthenogenesis*
	27	162 162	*103 115*	157 157	192 192	284 286	89 97	121 121	135 135	- -	*parthenogenesis*
	28	162 162	*103 115*	157 157	192 192	284 286	89 97	121 121	135 135	- -	*parthenogenesis*
	29	162 162	*103 115*	157 157	192 192	284 286	89 97	121 121	135 135	- -	*parthenogenesis*
	30	162 162	*103 115*	157 157	192 192	284 286	89 97	121 121	135 135	- -	*parthenogenesis*
	31	162 162	*103 115*	157 157	192 192	284 286	89 89	121 121	135 135	- -	*parthenogenesis*
	32	162 162	*103 115*	157 157	192 192	286 286	89 89	121 121	135 135	- -	*parthenogenesis*
	33	162 162	*103 115*	157 157	192 192	284 286	89 97	121 121	135 135	- -	*parthenogenesis*
	34	162 162	*103 115*	157 157	192 192	284 286	89 97	121 121	135 135	- -	*parthenogenesis*
	35	162 162	*103 115*	157 157	192 192	284 286	89 97	121 121	135 135	- -	*parthenogenesis*
	36	162 162	*103 115*	157 157	192 192	284 286	*97 97*	121 121	135 135	- -	*parthenogenesis*
	37	162 162	*103 115*	157 157	192 192	284 286	89 97	121 121	135 135	- -	*parthenogenesis*
	38	162 162	*103 115*	157 157	192 192	284 286	89 97	121 121	135 135	- -	*parthenogenesis*
	39	162 162	*103 115*	157 157	192 192	284 286	89 97	121 121	135 135	- -	*parthenogenesis*
	40	162 162	*115 115*	157 157	192 192	284 286	89 97	121 121	135 135	- -	*parthenogenesis*
	41	162 162	*103 115*	157 157	192 192	284 286	89 89	121 121	135 135	- -	*parthenogenesis*
	42	162 162	*103 115*	157 157	192 192	284 284	*97 97*	121 121	135 135	- -	*parthenogenesis*
	43	162 162	*103 115*	157 157	192 192	284 286	89 97	121 121	135 135	- -	*parthenogenesis*
	44	162 162	103 **111**	157 **167**	192 192	284 286	89 89	121 121	135 135	- -	*sex*
**new neotenic queens**<br>10	1	162 162	*103 115*	157 157	192 192	284 286	89 97	121 121	135 135	- -	*parthenogenesis*
	2	162 162	*103 115*	157 157	192 192	284 286	89 97	121 121	135 135	- -	*parthenogenesis*
	3	162 162	*103 115*	157 157	192 192	284 286	89 97	121 121	135 135	- -	*parthenogenesis*
	4	162 162	*103 115*	157 157	192 192	284 286	89 97	121 121	135 135	- -	*parthenogenesis*
	5	162 162	*103 115*	157 157	192 192	284 286	89 89	121 121	135 135	- -	*parthenogenesis*
	6	162 162	*103 115*	157 157	192 192	284 286	89 97	121 121	135 135	- -	*parthenogenesis*
	7	162 162	*103 115*	157 157	192 192	284 286	89 97	121 121	135 135	- -	*parthenogenesis*
	8	162 162	*103 115*	157 157	192 192	284 286	89 97	121 121	135 135	- -	*parthenogenesis*
	9	162 162	*103 115*	157 157	192 192	286 286	89 97	121 121	135 135	- -	*parthenogenesis*
	10	162 162	*103 115*	157 157	192 192	284 286	89 97	121 121	135 135	- -	*parthenogenesis*

* - due to technical failure, the marker En-39 did not amplify in Colony H

COLONY I

Caste n		En-8	En-10	En-11	En-15	En-19	En-25	En-35	En-37	En-39	genetic origin
**primary queen** *(inferred)*		157 157	113 113	162 162	286 286	190 194	89 97	119 119	135 135	158 164	
**primary king** *(inferred)*		157 **169**	113 113	162 162	**294** **292**	**178** 190	89 97	119 119	**131** **147**	158 158	
**workers** 14		157 157 *(6)*	113 113 *(14)*	162 162 *(14)*	286 **292** *(7)*	190 190 *(3)*	89 89 *(5)*	119 119 *(14)*	**131** 135 *(9)*	158 158 *(6)*	
		157 **169** *(8)*			286 **294** *(7)*	**178** 190 *(3)*	89 97 *(5)*		135 **147** *(5)*	158 164 *(8)*	
						**178** 194 *(4)*	97 97 *(4)*				
						190 194 *(4)*					
**soldiers** 9		157 157 *(7)*	113 113 *(9)*	162 162 *(9)*	286 **292** *(3)*	190 190 *(2)*	89 89 *(3)*	119 119 *(9)*	**131** 135 *(4)*	158 158 *(4)*	
		157 **169** *(2)*			286 **294** *(6)*	**178** 190 *(2)*	89 97 *(3)*		135 **147** *(5)*	158 164 *(5)*	
						**178** 194 *(3)*	97 97 *(3)*				
						190 194 *(2)*					
**nymphs (NY4♀)** 65	1	157 157	113 113	162 162	*286 286*	190 194	89 97	119 119	*135 135*	158 164	*parthenogenesis*
	2	157 157	113 113	162 162	*286 286*	190 194	89 97	119 119	*135 135*	158 164	*parthenogenesis*
	3	157 157	113 113	162 162	*286 286*	190 194	89 97	119 119	*135 135*	158 164	*parthenogenesis*
	4	157 157	113 113	162 162	*286 286*	190 194	89 97	119 119	*135 135*	158 164	*parthenogenesis*
	5	157 157	113 113	162 162	*286 286*	190 194	89 97	119 119	*135 135*	158 164	*parthenogenesis*
	6	157 157	113 113	162 162	*286 286*	190 194	97 97	119 119	*135 135*	158 164	*parthenogenesis*
	7	157 157	113 113	162 162	*286 286*	190 194	89 89	119 119	*135 135*	158 164	*parthenogenesis*
	8	157 157	113 113	162 162	*286 286*	190 194	89 97	119 119	*135 135*	158 164	*parthenogenesis*
	9	157 157	113 113	162 162	*286 286*	190 194	89 97	119 119	*135 135*	158 164	*parthenogenesis*
	10	157 157	113 113	162 162	*286 286*	190 194	89 97	119 119	*135 135*	158 164	*parthenogenesis*
	11	157 157	113 113	162 162	*286 286*	190 194	89 97	119 119	*135 135*	158 164	*parthenogenesis*
	12	157 157	113 113	162 162	*286 286*	190 194	89 97	119 119	*135 135*	158 164	*parthenogenesis*
	13	157 157	113 113	162 162	*286 286*	190 194	89 97	119 119	*135 135*	158 164	*parthenogenesis*
	14	157 157	113 113	162 162	*286 286*	190 194	89 89	119 119	*135 135*	*164 164*	*parthenogenesis*
	15	157 157	113 113	162 162	*286 286*	*194 194*	89 89	119 119	*135 135*	*164 164*	*parthenogenesis*
	16	157 157	113 113	162 162	*286 286*	190 194	97 97	119 119	*135 135*	158 164	*parthenogenesis*
	17	157 157	113 113	162 162	*286 286*	190 194	89 97	119 119	*135 135*	158 164	*parthenogenesis*
	18	157 157	113 113	162 162	*286 286*	190 194	89 97	119 119	*135 135*	158 164	*parthenogenesis*
	19	157 157	113 113	162 162	*286 286*	*194 194*	89 97	119 119	*135 135*	*164 164*	*parthenogenesis*
	20	157 157	113 113	162 162	*286 286*	190 194	89 97	119 119	*135 135*	158 164	*parthenogenesis*
	21	157 157	113 113	162 162	*286 286*	190 194	97 97	119 119	*135 135*	158 164	*parthenogenesis*
	22	157 157	113 113	162 162	*286 286*	190 194	89 97	119 119	*135 135*	158 164	*parthenogenesis*
	23	157 157	113 113	162 162	*286 286*	190 190	89 97	119 119	*135 135*	158 164	*parthenogenesis*
	24	157 157	113 113	162 162	*286 286*	190 194	89 97	119 119	*135 135*	158 164	*parthenogenesis*
	25	157 157	113 113	162 162	*286 286*	190 194	89 97	119 119	*135 135*	158 164	*parthenogenesis*
	26	157 157	113 113	162 162	*286 286*	190 194	89 97	119 119	*135 135*	158 164	*parthenogenesis*
	27	157 157	113 113	162 162	*286 286*	190 194	89 89	119 119	*135 135*	158 164	*parthenogenesis*
	28	157 157	113 113	162 162	*286 286*	190 194	89 97	119 119	*135 135*	158 164	*parthenogenesis*
	29	157 157	113 113	162 162	*286 286*	190 194	97 97	119 119	*135 135*	158 164	*parthenogenesis*
	30	157 157	113 113	162 162	*286 286*	190 194	89 97	119 119	*135 135*	158 164	*parthenogenesis*
	31	157 157	113 113	162 162	*286 286*	190 194	97 97	119 119	*135 135*	158 164	*parthenogenesis*
	32	157 157	113 113	162 162	286 **294**	**178** 190	97 97	119 119	**131** 135	158 158	*sex*
	33	157 **169**	113 113	162 162	286 **294**	**178** 194	89 97	119 119	135 **147**	158 158	*sex*
	34	157 157	113 113	162 162	286 **294**	190 190	89 97	119 119	**131** 135	158 158	*sex*
	35	157 **169**	113 113	162 162	286 **292**	**178** 190	89 97	119 119	**131** 135	158 164	*sex*
	36	157 157	113 113	162 162	286 **292**	190 190	89 89	119 119	135 **147**	158 164	*sex*
	37	157 **169**	113 113	162 162	286 **294**	**178** 194	89 97	119 119	**131** 135	158 158	*sex*
	38	157 157	113 113	162 162	286 **292**	**178** 194	89 97	119 119	**131** 135	158 158	*sex*
	39	157 157	113 113	162 162	286 **294**	190 190	97 97	119 119	135 **147**	158 164	*sex*
	40	157 **169**	113 113	162 162	286 **294**	190 194	97 97	119 119	**131** 135	158 164	*sex*
	41	157 157	113 113	162 162	286 **294**	190 190	97 97	119 119	**131** 135	158 158	*sex*
	42	157 **169**	113 113	162 162	286 **294**	**178** 194	89 97	119 119	**131** 135	158 164	*sex*
	43	157 157	113 113	162 162	286 **292**	**178** 190	89 97	119 119	**131** 135	158 158	*sex*
	44	157 **169**	113 113	162 162	286 **292**	**178** 194	97 97	119 119	**131** 135	158 158	*sex*
	45	157 157	113 113	162 162	286 **292**	190 190	89 97	119 119	**131** 135	158 164	*sex*
	46	157 **169**	113 113	162 162	286 **294**	**178** 194	89 97	119 119	135 **147**	158 164	*sex*
	47	157 **169**	113 113	162 162	286 **294**	**178** 190	97 97	119 119	**131** 135	158 158	*sex*
	48	157 157	113 113	162 162	286 **292**	190 190	89 89	119 119	135 **147**	158 164	*sex*
	49	157 **169**	113 113	162 162	286 **292**	190 194	89 97	119 119	**131** 135	158 158	*sex*
	50	157 157	113 113	162 162	286 **292**	190 194	89 97	119 119	135 **147**	158 164	*sex*
	51	157 **169**	113 113	162 162	286 **292**	**178** 190	97 97	119 119	**131** 135	158 158	*sex*
	52	157 **169**	113 113	162 162	286 **294**	**178** 190	89 97	119 119	135 **147**	158 164	*sex*
	53	157 **169**	113 113	162 162	286 **292**	**178** 194	89 89	119 119	135 **147**	158 164	*sex*
	54	157 **169**	113 113	162 162	286 **292**	190 190	89 89	119 119	135 **147**	158 164	*sex*
	55	157 **169**	113 113	162 162	286 **294**	**178** 194	97 97	119 119	**131** 135	158 158	*sex*
	56	157 157	113 113	162 162	286 **292**	**178** 190	97 97	119 119	**131** 135	158 164	*sex*
	57	157 **169**	113 113	162 162	286 **292**	190 194	89 97	119 119	**131** 135	158 164	*sex*
	58	157 **169**	113 113	162 162	286 **292**	190 190	97 97	119 119	**131** 135	158 164	*sex*
	59	157 157	113 113	162 162	286 **292**	190 194	97 97	119 119	**131** 135	158 164	*sex*
	60	157 **169**	113 113	162 162	286 **292**	190 194	89 89	119 119	**131** 135	158 158	*sex*
	61	157 **169**	113 113	162 162	286 **294**	**178** 194	89 97	119 119	135 **147**	158 158	*sex*
	62	157 157	113 113	162 162	286 **292**	190 190	97 97	119 119	**131** 135	158 164	*sex*
	63	157 **169**	113 113	162 162	286 **294**	**178** 190	89 97	119 119	**131** 135	158 164	*sex*
	64	157 **169**	113 113	162 162	286 **294**	**178** 190	89 97	119 119	135 **147**	158 164	*sex*
	65	157 157	113 113	162 162	286 **294**	**178** 190	89 97	119 119	135 **147**	158 164	*sex*
**new neotenic queens** 7	1	157 **169**	113 113	162 162	286 **294**	**178** 190	89 97	119 119	135 **147**	158 164	*sex*
	2	157 157	113 113	162 162	*286 286*	190 194	89 97	119 119	*135 135*	158 164	*parthenogenesis*
	3	157 157	113 113	162 162	*286 286*	190 194	97 97	119 119	*135 135*	158 164	*parthenogenesis*
	4	157 157	113 113	162 162	*286 286*	190 194	97 97	119 119	*135 135*	158 164	*parthenogenesis*
	5	157 157	113 113	162 162	*286 286*	190 194	97 97	119 119	*135 135*	158 164	*parthenogenesis*
	6	157 157	113 113	162 162	*286 286*	190 194	89 89	119 119	*135 135*	158 164	*parthenogenesis*
	7	157 157	113 113	162 162	*286 286*	190 194	89 97	119 119	*135 135*	158 164	*parthenogenesis*

Supplementary Table 2. Primers and multiplexes used for genotyping.

Locus	Motif	Primer sequence (5'-3')	Label
Multiplex 1			
En11	$(ac)_{14}$	F: CCAACTCGTAGGTGTAGAGGAT<br>R: CCGTCTCTTGTGAGTGTTGTG	NED
En10	$(tg)_{14}$	F: CGTCCAGAAGATTCCTACCG<br>R: TCTCTACCTCGTGTCTGCCT	NED
En08	$(ac)_{13}$	F: CTGAGCGGTTGCAGAGTACC<br>R: TTCCCGGCCAAAGTACTAAC	6'FAM
En19	$(tg)_{17}$	F: TACATTCAAATTAGTCTTGTGCCC<br>R: TTGGTCGAGCCTATCTGGTC	PET
En15	$(ca)_{15}$	F: CGATGAGATTCCGTAGACACC<br>R: AACCCTAGCACCTCACATGC	NED
Multiplex 2			
En25	$(ac)_{11}$	F: AGTTCGCGTTCAGAAGAAGC<br>R: TTCTCAATCAATGCAACCTGTC	NED
En35	$(ca)_{09}$	F: ACAGAGTGGCCTCTTTACGC<br>R: CCCATTCAAGCACGTCTGTA	VIC
En37	$(ac)_{09}$	F: ACGCGCACAGTATTGCAT<br>R: TGAGTGTGGTGGGGTAATGT	6'FAM
En39	$(ag)_{09}$	F: GCTTCCAGTGTAAATCACAATTTC<br>R: GCAGTGAGATTTGTAGCCCC	PET

SUPPLEMENTARY REFERENCES

1. Fougeyrollas, R. *et al.* Asexual queen succession in the higher termite *Embiratermes neotenicus*. *Proc. R. Soc. B* **282**, 20150260 (2015).

2. Fougeyrollas, R. *et al.* Dispersal and mating strategies in two neotropical soil-feeding termites, *Embiratermes neotenicus* and *Silvestritermes minutus* (Termitidae, Syntermitinae). *Insectes Soc.* **65**, 251–262 (2018).
