# Supplementary material for: Identification of a queen primer pheromone in higher termites
Source: Commun Biol. 2022 Nov 2;5:1165. doi: 10.1038/s42003-022-04163-5 (PMC9630296; doi:10.1038/s42003-022-04163-5)
Supplement: Supplementary file 2 — Description of Additional Supplementary Files [file 42003_2022_4163_MOESM2_ESM.pdf]

Description of Additional Supplementary Files

File name: Supplementary Data 1
Description: The source data behind the graphs and underlying statistics in the main text.
