# Supplementary material for: Identification of a queen primer pheromone in higher termites
Source: Commun Biol. 2022 Nov 2;5:1165. doi: 10.1038/s42003-022-04163-5 (PMC9630296; doi:10.1038/s42003-022-04163-5)
Supplement: Supplementary file 4 — Reporting Summary file [file 42003_2022_4163_MOESM4_ESM.pdf]

nature portfolio

Corresponding author(s): Robert Hanus

Last updated by author(s): Oct 17, 2022

Reporting Summary

Nature Portfolio wishes to improve the reproducibility of the work that we publish. This form provides structure for consistency and transparency in reporting. For further information on Nature Portfolio policies, see our Editorial Policies and the Editorial Policy Checklist.

Statistics

For all statistical analyses, confirm that the following items are present in the figure legend, table legend, main text, or Methods section.

n/a	Confirmed	
☐	☒	The exact sample size (*n*) for each experimental group/condition, given as a discrete number and unit of measurement
☐	☒	A statement on whether measurements were taken from distinct samples or whether the same sample was measured repeatedly
☐	☒	The statistical test(s) used AND whether they are one- or two-sided<br>*Only common tests should be described solely by name; describe more complex techniques in the Methods section.*
☐	☒	A description of all covariates tested
☐	☒	A description of any assumptions or corrections, such as tests of normality and adjustment for multiple comparisons
☐	☒	A full description of the statistical parameters including central tendency (e.g. means) or other basic estimates (e.g. regression coefficient) AND variation (e.g. standard deviation) or associated estimates of uncertainty (e.g. confidence intervals)
☐	☒	For null hypothesis testing, the test statistic (e.g. *F*, *t*, *r*) with confidence intervals, effect sizes, degrees of freedom and *P* value noted<br>*Give P values as exact values whenever suitable.*
☒	☐	For Bayesian analysis, information on the choice of priors and Markov chain Monte Carlo settings
☒	☐	For hierarchical and complex designs, identification of the appropriate level for tests and full reporting of outcomes
☒	☐	Estimates of effect sizes (e.g. Cohen's *d*, Pearson's *r*), indicating how they were calculated

Our web collection on statistics for biologists contains articles on many of the points above.

Software and code

Policy information about availability of computer code

Data collection	All calculations were performed and graphs generated in GraphPad Prism v 8.0 (GraphPad Software Inc., San Diego, CA). Electrophysiological data has been collected and digitalized, and maximal negative deflection quantified using Syntech EAG software (Ockenfels SYNTECH GmbH, Buchenbach, Germany).
Data analysis	All calculations were performed and graphs generated in GraphPad Prism v 8.0 (GraphPad Software Inc., San Diego, CA).

For manuscripts utilizing custom algorithms or software that are central to the research but not yet described in published literature, software must be made available to editors and reviewers. We strongly encourage code deposition in a community repository (e.g. GitHub). See the Nature Portfolio guidelines for submitting code & software for further information.

Data

Policy information about availability of data

All manuscripts must include a data availability statement. This statement should provide the following information, where applicable:

- Accession codes, unique identifiers, or web links for publicly available datasets
- A description of any restrictions on data availability
- For clinical datasets or third party data, please ensure that the statement adheres to our policy

Data generated or analyzed during this study are included in Supplementary Data file and in Supplementary information file.

Human research participants

Policy information about studies involving human research participants and Sex and Gender in Research.

Reporting on sex and gender	Not applicable.
Population characteristics	Not applicable.
Recruitment	Not applicable.
Ethics oversight	Not applicable.

Note that full information on the approval of the study protocol must also be provided in the manuscript.

Field-specific reporting

Please select the one below that is the best fit for your research. If you are not sure, read the appropriate sections before making your selection.

☒ Life sciences ☐ Behavioural & social sciences ☐ Ecological, evolutionary & environmental sciences

For a reference copy of the document with all sections, see nature.com/documents/nr-reporting-summary-flat.pdf

Life sciences study design

All studies must disclose on these points even when the disclosure is negative.

Sample size	EXPERIMENT I:<br>Selection of the number of colonies used: Three colonies per one demographic category (dispersing vs. non-dispersing colony) were selected as sufficient and optimal to show absence vs. presence of the phenomenon (presence/absence of queen differentiation).<br>Sample size within each colony: The number of nymphs in the experiment has been set to 17, since it was the lowest number of female nymphs (NY4) found in an individual colony.<br><br>EXPERIMENT II and III:<br>To demonstrate the effect of the studied treatment (EXP II) and explore the range of potentially effective doses we selected one colony as the proof of principle object.<br>To further confirm the observations from EXP II and study the role of the treatment as an airborne stimulus, two other colonies were selected, and treatment doses were selected based on the output of EXP II.<br>Confirmation of pheromone effects on three colonies is a golden standard in chemical ecology of social insects.<br><br>Five replicates per each treatment from each colony were used to allow sufficient statistical power and assess possible group effects in experimental groups.<br><br>Sample size (number of nymphs) per replicate was set to nine since it was the number of nymphs available for all treatments and replicates in the first studied colony (EXP II).<br><br>Two independent electrophysiological experiments were performed, one with 50 ng and the other with 500 ng of four compounds. In each experiment, 15 antennae of 15 different nymphs were stimulated with a series of the four stimuli in randomized order. The number of replicates has been selected as a maximum possible given the availability of nymphs in the studied colony, and deemed by far sufficient.
Data exclusions	We did not exclude any data a posteriori.
Replication	The study consists of independent replications at multiple levels: 10 independent colonies were used, 3 independent colonies were used for the main experiment, each treatment consisted of 3 replicates. See above (Sample size) for details. Attempts at replication were successful.
Randomization	Randomization was performed at multiple levels: 1) All nymphs selected in individual colonies were randomly distributed among replicate groups. 2) In electrophysiological experiments, the stimulations were randomly ordered in each stimulation series.
Blinding	Regular census of individuals (EXP I-III, every 6 hours) was performed independently by two censors.

Reporting for specific materials, systems and methods

We require information from authors about some types of materials, experimental systems and methods used in many studies. Here, indicate whether each material, system or method listed is relevant to your study. If you are not sure if a list item applies to your research, read the appropriate section before selecting a response.

Materials & experimental systems

n/a	Involved in the study
☒	☐ Antibodies
☒	☐ Eukaryotic cell lines
☒	☐ Palaeontology and archaeology
☐	☒ Animals and other organisms
☒	☐ Clinical data
☒	☐ Dual use research of concern

Methods

n/a	Involved in the study
☒	☐ ChIP-seq
☒	☐ Flow cytometry
☒	☐ MRI-based neuroimaging

Animals and other research organisms

Policy information about studies involving animals; ARRIVE guidelines recommended for reporting animal research, and Sex and Gender in Research

Laboratory animals	The study did not involve any.
Wild animals	Mature colonies of Embiratermes neotenicus (Termitidae: Syntermitinae) were collected in the field (forests of French Guiana).
Reporting on sex	Not applicable.
Field-collected samples	All colonies but one were used for experiments performed at a research station in French Guiana within four days of their collection. The colonies were then returned to the original forest location. One colony was transported in a plastic jar to the laboratory in Prague (Europe). The colony was kept in the laboratory at 27°C in a glass aquarium with controlled humidity.
Ethics oversight	Under current legislation no ethical approval is required.

Note that full information on the approval of the study protocol must also be provided in the manuscript.
